# Mode-dependent effects of chemical, intermittent, and hypobaric hypoxia on HIF-1α, HIF-2α, HIF-3α, PACAP, and PAI mRNA expression in the single prolonged stress model of PTSD

**DOI:** 10.3389/fnins.2026.1904848

**Published:** 2026-07-31

**Authors:** Denys Porkhalo, Denys Pashevin, Yana Naumenko, Roman Koval, Mariia Kozlovska, Mariia Kamkina, Yan Tytarenko, Alla Portnychenko, Victor Dosenko

**Affiliations:** 1 Department of General and Molecular Pathophysiology, Bogomoletz Institute of Physiology of NAS of Ukraine, Kyiv, Ukraine; 2 Department of Biophysics of Sensory Signalling, Bogomoletz Institute of Physiology of NAS of Ukraine, Kyiv, Ukraine; 3 Department of Cytology, Bogomoletz Institute of Physiology of NAS of Ukraine, Kyiv, Ukraine; 4 Department of Hypoxia, Bogomoletz Institute of Physiology of NAS of Ukraine, Kyiv, Ukraine; 5 International Centre for Astronomical and Medico-Ecological Research, NAS of Ukraine, Kyiv, Ukraine; 6 Kyiv Medical University, Kyiv, Ukraine; 7 Bogomolets National Medical University, Kyiv, Ukraine

**Keywords:** fear conditioning, HIF-1α/2α/3α, hypoxia, PACAP, PAI, post-traumatic stress disorder

## Abstract

**Introduction:**

Although hypoxic interventions are increasingly explored as non-pharmacological modulators of stress-related pathology, their ability to benefit PTSD remains poorly understood. We compared behavioural and molecular outcomes of three hypoxic regimes — intermittent normobaric hypoxia (IHT), hypobaric hypoxia (Bar), and chemical hypoxia (CH) via cobalt chloride (CoCl₂) — in rats with PTSD induced by single prolonged stress (SPS).

**Methods:**

Male albino rats (*n* = 105; eight groups) received one of the three treatments after control or SPS conditioning. Anxiety-like behaviour was assessed by Elevated Plus Maze (EPM), Open Field Test (OFT), and Dark-Light Box (DLB); HIF-1α, HIF-2α, HIF-3α, PACAP, and PAI-1 mRNA were quantified in the medial prefrontal cortex (mPFC) and hippocampus by real-time qPCR.

**Results:**

Mild IHT was the only regimen to produce robust PTSD-specific anxiolysis: PTSD+IHT animals showed a four-fold reduction in EPM freezing relative to Control+IHT (5.4 ± 4.9 vs 21.6 ± 14.9 s; *p* < 0.01), a near four-fold reduction in OFT freezing (30.3 ± 8.6 vs 119.9 ± 46.3 s; *p* < 0.001), and greater open-arm engagement. CH and Bar produced limited or no behavioural benefit. All three regimes substantially reduced HIF-1α mRNA and strongly reduced PAI-1 (>20-fold in most groups; *p* ≤ 0.001) irrespective of trauma status. Only HIF-1α was significantly elevated in the hippocampus of untreated PTSD animals relative to controls (2.78 ± 1.97 vs 1.10 ± 0.50; *q* = 0.039); HIF-2α and HIF-3α showed a similar, non-significant upward trend (both ns after FDR correction, reflecting wide between-animal variability). Group means for all three HIF subunits converged toward the control range under all three interventions. HIF-3α responses to IHT or Bar were consistently blunted in PTSD animals across both regions, while PACAP was uniformly suppressed with stronger trauma-dependent attenuation under Bar.

**Discussion:**

As protein-level and canonical HIF target-gene readouts were not assessed, the reported transcript changes should be interpreted as a downstream feedback signature rather than a direct index of functional HIF activity. Hypoxic modality, not hypoxia per se, determines behavioural and molecular responses in PTSD; mild IHT uniquely normalised both in traumatised animals, supporting its development as a state-dependent non-pharmacological intervention for trauma-related conditions.

## Introduction

PTSD is one of the most common pathologies in the general population (particularly in countries experiencing armed conflict) (Charlson et al., 2019). The study of this disorder requires a multifaceted examination of its behavioural, physiological, and molecular aspects.

Development or compensation of stress may be associated with multiple hypoxia-dependent ways. In a related rodent paradigm—a learned-helplessness/footshock model of stress-induced depression rather than the single-prolonged-stress (SPS) model used here—psychoemotional stress was shown to cause prolonged overexpression of HIF-1α in the hippocampus and the neocortex of rats (Rybnikova et al., 2015). In contrast, changes in oxygen homeostasis can have positive effects on patients with PTSD. For example, stay in a hyperbaric chamber alleviated PTSD symptoms in veterans (Doenyas-Barak et al., 2024). Intermittent hypoxia has been shown to affect brain health positively (Boulares et al., 2024). However, different hypoxia regimes exhibited different or opposite effects: intermittent hypoxia decreased BDNF blood levels in young adults when applied for a long term (Becke et al., 2018), or stimulated this factor after short-term exposure (Vermehren-Schmaedick et al., 2012). In the same line of work, hypoxic pre- and postconditioning with mild hypobaric hypoxia abolished the prolonged HIF-1α overexpression in the hippocampus and promoted adaptation to psychoemotional stress, although in a stress model distinct from SPS (Rybnikova et al., 2015). Similar positive effects against stress-related anxiety disorders were also induced by remote ischemic pre- and postconditioning (Zhao et al., 2017; Li et al., 2025). These and other observations indicate the need for detailed study of the effects of different hypoxia regimes in PTSD.

Currently, the use of intermittent hypoxic training/conditioning as a non-pharmacological therapy is a recognised approach to increasing the body’s resistance to extreme factors and health benefits (see Serebrovskaya and Xi, 2016; Burtscher et al., 2024 for reviews). Amongst other well-known approaches including “lifting” in barochambers, hypoxic gas mixture breathing or with rebreathing devices, we were interested in the testing of hypobaric hypoxia, particularly more intense regimens.

So called “chemical hypoxia” (CH) induced by cobalt chloride (CoCl₂) is also used in animal models (Muñoz-Sánchez and Chánez-Cárdenas, 2019). CoCl₂ mimics hypoxia by inhibiting Fe^2+^-dependent dioxygenases, and it exhibits the greatest activity against PHD and FIH-1—the enzymes that regulate the stability and transcriptional activity of HIFs (Muñoz-Sánchez and Chánez-Cárdenas, 2019). In this way, CH activates HIF-1α and downstream ways in cell and animal models (Yuan et al., 2003). It was demonstrated that preconditioning and post-treatment with CoCl₂ protects against brain damage in rat model of perinatal hypoxic–ischemic encephalopathy (Dai et al., 2014). However, effect of CH on PTSD-like animal models is unknown and needs to be studied. At the same time, the study of CH allows to identify amongst the mechanisms of influence the pathways associated with the stabilisation of HIFs. Comparing CH with hypoxic exposures therefore allows dissecting which downstream mechanisms depend on HIF stabilisation per se and which require additional stimuli. The role of HIF subunits also needs to be clarified.

Within these goals, several other molecules are of interest: plasminogen activator inhibitor-1 (PAI-1) and pituitary adenylate cyclase-activating polypeptide (PACAP). PAI-1 indirectly inhibits brain-derived neurotrophic factor (BDNF) maturation by blocking tissue plasminogen activator (tPA), thereby reducing plasmin-mediated cleavage of proBDNF (Bouarab et al., 2021; Pang et al., 2004). Conversely, PACAP, a neuropeptide, has been shown to modulate neuroinflammation and neuronal plasticity, suggesting a potential role in counteracting the pathogenic mechanisms observed in PTSD (Ressler et al., 2011; Gilmartin and Ferrara, 2021; Hashimoto et al., 2011; Stroth et al., 2011). It has also been shown that PACAP, in turn, can not only modulate PAI-1 activity but also act as a peptide with a dual role: it can be neuroprotective or exacerbate neurodegenerative symptoms (Riser and Norrholm, 2022). Given these interactions, understanding the contribution of PAI-1 and PACAP in hypoxia-associated response may be important for developing targeted therapeutic strategies in stress-induced psychopathologies, such as PTSD.

We investigated the molecular pathways involved in the development and symptoms of SPS-induced PTSD in rats exposed to different hypoxia regimens, aiming to compare their effects on behavioural traits. This approach combines behavioural data with molecular studies to better understand this disorder.

## Materials and methods

### Animals

Experiments were performed in 105 three-month-old male albino rats (*Rattus norvegicus*) weighing 200–250 g, obtained from the vivarium of the Bogomoletz Institute of Physiology, NAS of Ukraine. Animals were group-housed with water and food available *ad libitum*. All experimental stages were performed according to the law of Ukraine “On the Protection of Animals from Cruelty” (Article 26; No 3447—IV) and European Convention for the Protection of Vertebrate Animals used for Experimental and Other Scientific Purposes. Strasbourg: Council of Europe, European Treaty Series No. 123 (1986). We did everything we could to minimise the animals’ suffering and use fewer of them. The rats were randomly divided into eight groups: (1) Control (*n* = 18)—rats without any interventions; (2) Сontrol + CoCl_2_ (*n* = 12)—rats with application of CoCl_2_; (3) Сontrol + IHT (*n* = 12)—rats with intermittent normobaric hypoxia treatment; (4) Control + Bar (*n* = 12)—rats with hypobaric hypoxia treatment; (5) PTSD (*n* = 18)—rats with SPS-induced PTSD; (6) PTSD + CoCl_2_ (*n* = 11)—PTSD rats with application of CoCl_2_; (7) PTSD + IHT (*n* = 10)—PTSD rats with intermittent normobaric hypoxia treatment; (8) PTSD + Bar (*n* = 12)—PTSD rats with hypobaric hypoxia treatment. Three animals died during SPS induction and were excluded; additional animals were excluded *post hoc* as drop-outs because of insufficient brain tissue at dissection or aggressive behaviour towards cage-mates that precluded group housing, accounting for the variation in final group sizes.

Animals were housed under standard vivarium conditions and allowed a 7-day acclimatisation period before experimental procedures. During acclimatisation, animals had ad libitum access to food and water and were maintained under a 12-h light/dark cycle. Rats were transferred from the institute vivarium to a separate experimental room for acclimatisation to the new housing conditions and handling before experimental procedures.

### Single prolonged stress

The SPS protocol was used for PTSD-like phenotype induction in rats (Liberzon et al., 1997; Lisieski et al., 2018). SPS was conducted as a single-day procedure with three sequential stressors. First, animals were subjected to immobiliation stress for 120 min by placing them in a restrainer whilst allowing them to breathe normally. Immediately after immobiliation, animals were subjected to forced swimming in room-temperature water (22 ± 1 °C) for 15 min in individual cylinders. Following a brief drying period, animals were exposed to diethyl ether vapour in a sealed induction chamber until they lost the righting reflex (approximately 30–60 s). Throughout the ether exposure, animals were monitored continuously to ensure unconsciousness without respiratory arrest. After recovering from anaesthesia, animals were returned to their home cages and left undisturbed for 7 days (incubation period) before any further experimental procedures.

### Hypoxic interventions

*Intermittent normobaric hypoxia (IHT)* was reproduced by exposing animals to breathing with controlled hypoxic gas mixture at atmospheric pressure. Groups of five animals were placed in sealed glass chambers consecutively blowing with hypoxic gas mixture of 14% oxygen and 86% nitrogen (рO_2_ = 106 mmHg, calculated as 0.14 × 760 mmHg at normobaric pressure; equivalent to breathing ambient air at ~3,300 m altitude (barometric pressure ~505 Torr)) or with room air, supplied at a flow rate sufficient to saturate the chamber during the first minute of each exposure cycle. Each intermittent hypoxic exposure cycle consisted of 5 min of hypoxia followed by 5 min of normoxia (room air), with cycles repeated continuously over a total session duration of 60 min (6 complete cycles per session with an initial 1-min chamber equilibration period). Sessions were conducted once daily for 14 consecutive days.

*Hypobaric hypoxia (Bar)* was performed by “lifting” rats in barochamber to a simulated altitude up to 4,500 m (440 Torr, рO_2_ = 92 mmHg (dry ambient; humidified inspired pO₂ ≈ 82 mmHg)). The intermittent hypobaric exposure paradigm (4,500 m; 4 h/day) was adapted from the model described by Santocildes et al. (2021); at 4,500 m an inspired partial pressure of oxygen is of ~82 mmHg giving a moderate-to-severe hypoxic stimulus. Therefore, gradual ascent over 1–5 days was used as an additional acclimatisation procedure to minimise additional hypoxic stress in the animals. The acclimatisation protocol was as follows: Day 1–1,000 m simulated altitude (676 Torr) for 30 min; Day 2–2,000 m (600 Torr) for 60 min; Day 3–3,000 m (532 Torr) for 120 min; Day 4–4,000 m (463 Torr) for 180 min; Day 5–4,500 m (440 Torr) for 240 min. From Day 6 through Day 14, animals were exposed daily to a simulated altitude of 4,500 m (440 Torr) for 240 min per session.

*Chemical hypoxia (CH)* was simulated with cobalt chloride (CoCl_2_) administration. CoCl_2_ was dissolved in distilled water and administered orally via gavage as 0.1 mL of solution at a dose of 15 mg/kg body weight once daily for seven consecutive days. The CoCl₂ regimen was deliberately limited to seven days, in contrast to the 14-day IHT and Bar protocols, because cobalt chloride is cumulatively toxic and prolonged administration carries a substantial risk of systemic and organ toxicity; the shorter schedule was therefore chosen to maintain HIF stabilisation whilst avoiding the adverse effects associated with extended exposure (Jomova and Valko, 2011; Chen et al., 2019).

### Behavioural tests

Behavioural assessments were conducted 7 days after SPS, during light hours (09:00–15:00), in a controlled room at 22 ± 1 °C under warm lighting. Animals arrived 30 min early for acclimatisation. Tests were done in the following order: Open Field, Elevated Plus Maze, and Dark–Light Box, with at least 24 h between tests to reduce carry-over effects. The apparatus was cleaned with 70% ethanol and dried between animals to remove olfactory cues. All tests were video-recorded and analysed with AnyMaze software (Stoelting Co., USA).

#### Open Field

Locomotor activity and anxiety-like behaviour were assessed in an arena (75 × 75 × 40 cm, grey PVC). Each animal was placed in the centre and explored for 10 min. The arena had three zones: central, peripheral, and corners. Parameters recorded included total distance (m), mean speed (m/s), entries and time in each zone, distance in peripheral and corner zones, freezing episodes, and freezing time (s). Freezing was defined as no movement for ≥2 s.

#### Elevated Plus

The Elevated Plus Maze had two open and two enclosed arms (50 × 11 cm) connected by a central platform (11 × 11 cm), measuring 110 × 110 cm and 60 cm high. Each animal was placed on the central platform facing an open arm and observed for 5 min. Recorded parameters included total distance travelled, entries and time in open/closed arms, open-arm head dips (head over edge without body), rotations, freezing episodes, and total freezing time. Arm entry was defined as all four paws crossing into an arm.

#### Dark–Light Box

The apparatus had two compartments, each 56 × 56 × 40 cm: a lit one and a dark one connected by a 7 × 7 cm opening. The room light lit the compartment, whilst the dark was shielded. Each animal was placed in the dark, facing away from the opening, and allowed to explore freely for 5 min. Researchers recorded transitions between compartments, time in each, and curiosity behaviours, such as inserting the head into the lit compartment without full entry.

### Tissue collection

Animals were euthanized by decapitation following ketamine anaesthesia (100 mg/kg, i.p.). The hippocampus and medial prefrontal cortex (mPFC) were rapidly dissected on ice, snap-frozen in liquid nitrogen, and stored at −80 °C until further processing.

### RNA isolation

Total RNA was extracted using TRIzol reagent (Invitrogen, Thermo Fisher Scientific, USA) according to the manufacturer’s protocol. Briefly, frozen tissue was homogenised in 1 mL of TRIzol reagent, followed by phase separation with chloroform, precipitation with isopropanol, and washing with 75% ethanol. RNA pellets were resuspended in nuclease-free water and quantified spectrophotometrically. RNA purity was assessed by the A260/A280 absorbance ratio, with values between 1.8 and 2.0 considered acceptable for downstream analysis.

### Quantitative PCR

First-strand cDNA was synthesised from 1 μg of total RNA using the RevertAid First Strand cDNA Synthesis Kit (Thermo Fisher Scientific, USA) according to the manufacturer’s instructions, using random hexamer primers. DNase treatment was not performed before reverse transcription; amplification specificity was confirmed by melt curve analysis for all primer pairs, with single-peak melt curve profiles observed across all samples. No-reverse-transcriptase (no-RT) controls were included for each primer pair and showed no detectable amplification within the cycle range used for the corresponding RT(+) reactions, confirming the absence of genomic DNA contribution to the signal (Bustin et al., 2009).

Gene-specific primers for HIF1a, HIF-2α, HIF-3α, PACAP, and PAI-1 were designed for *Rattus norvegicus* target sequences. *β*-actin (Actb) was used as the reference gene for normalisation. All primers were synthesised by Yuria-Pharm (Ukraine) and validated for specificity by melt curve analysis before quantitative experiments. Amplicon sizes and the position of primer binding sites relative to exon–intron boundaries were verified using NCBI Primer-BLAST against the *Rattus norvegicus* genome (taxid: 10116) (Table 1); all primer pairs were designed as intron-spanning, with the forward and reverse primers annealing to different exons of the target transcript; combined with the absence of detectable amplification in no-RT controls, this design excludes a meaningful contribution of residual genomic DNA to the RT(+) signal. *β*-actin was selected as a reference gene on the basis of its established and widely used role as a housekeeping transcript; its expression stability under the present experimental conditions was not formally assessed using dedicated algorithms (e.g., geNorm or NormFinder).

**Table 1:** Primer sequences used for RT-qPCR analysis.

Gene	Protein	Forward primer (5′ → 3′)	Reverse primer (5′ → 3′)	Tm (°C)	Amplicon (bp)
*Hif1a*	*HIF-1α*	*GCAACTGCCACCACTGATGA*	*GCTGTCCGACTGTGAGTACC*	59.4/61.4	152
*Epas1*	*HIF-2α*	*ATGAGGCAGGACAGCAGGGG*	*GTCGGCCACCTGGAAGGTCT*	63.5/63.5	111
*Hif3a*	*HIF-3α*	*CGTGGATGTATTCATAGGCAGA*	*GCCTGGACATGAAGTTCACATA*	58.4/58.4	102
*Adcyap1*	*PACAP*	*GCCTCTCTGGTTGTGATTCCA*	*GGTCATTCGCGGCTAGGAA*	59.8/58.8	91
*Serpine1*	*PAI-1*	*CGTCTTCCTCCACAGCCATT*	*GTTGGATTGTGCCGAACCAC*	59.4/59.4	97
*Actb*	*β-actin*	*TCATCACTATCGGCAATGAGC*	*GGCCAGGATAGAGCCACCA*	57.9/61.0	302

For each target gene, the table lists the gene symbol, corresponding protein, forward and reverse primer sequences (5′ → 3′), melting temperature (Tm, °C), and expected amplicon size (bp). *β*-actin (Actb) was used as a reference gene for normalisation.

Real-time qPCR was performed on an Applied Biosystems 7,500 Fast Real-Time PCR System using iTaq Universal SYBR Green Supermix (Bio-Rad Laboratories, USA). Each reaction (10 μL total volume) contained 5 μL of SYBR Green supermix, 0.4 μL of each primer (10 pM), 1.5 μL of cDNA template, and 2.7 μL of nuclease-free water. The thermal cycling protocol consisted of an initial denaturation at 95 °C for 10 min, followed by 40 cycles of 95 °C for 15 s and 60 °C for 1 min, with a final melt curve stage (60–95 °C) to confirm amplicon specificity. All samples were run in duplicate, and a no-template control (NTC) was included on each plate. Relative gene expression was calculated using the 2^(−ΔCt) method (Livak and Schmittgen, 2001), with *β*-actin as the reference gene. Results are expressed as fold change relative to the Control group. All qPCR reactions were performed in singleplex, with β-actin and each target gene amplified in separate wells using identical thermal cycling; differences in total cDNA input between sample sets are corrected for independently for each transcript by the 2^(Ct_Actin − Ct_Gene) normalisation.

### Statistical analysis

Before statistical testing, outliers were identified and removed using the interquartile range (IQR) method, with values falling beyond 1.5 × IQR from the first or third quartile excluded from further analysis. The number of excluded observations per group is reported for each parameter.

Normality of distribution was assessed independently for each group using the Shapiro–Wilk test. When all groups met the normality criterion (*p* ≥ 0.05), between-group differences were evaluated using one-way analysis of variance (ANOVA) followed by an independent-samples t-test for pairwise comparisons. When at least one group deviated from normality, the Kruskal–Wallis test was applied as the omnibus test, with the Mann–Whitney U test used for post-hoc pairwise comparisons (two-tailed). Because the design involved more than two groups, a one-way omnibus test (ANOVA or Kruskal–Wallis) was applied before pairwise post-hoc comparisons; pairwise tests were restricted to twelve biologically predefined group pairs rather than all possible pairs.

For behavioural parameters (Open Field Test, Elevated Plus Maze, and Dark–Light Box), all comparisons between Control and PTSD groups were planned *a priori* based on the experimental design; accordingly, no correction for multiple comparisons was applied.

For gene expression data (HIF-1α, HIF-2α, HIF-3α, PACAP, and PAI-1 mRNA levels in cortex and hippocampus), *p*-values were adjusted for multiple comparisons using the Benjamini-Hochberg false discovery rate (FDR) procedure. Adjusted p-values (q-values) are reported alongside raw p-values where applicable. Each gene was treated as a separate hypothesis family (12 pairwise comparisons per gene), reflecting the rationale that each transcript represents an a priori independent biological endpoint.

Behavioural data are expressed as group medians with interquartile range; gene expression data are presented as relative concentration ([C]rel) calculated as 2^(Ct_Actin − Ct_Gene) × 100, where *β*-actin served as reference gene. All data are visualised as boxplots with individual data points overlaid. Significance thresholds were set at *p* < 0.05 (*), *p* < 0.01 (**), and *p* < 0.001 (***); non-significant comparisons are denoted as ns. All statistical analyses were performed in Python using the SciPy library (v. 1.11) and NumPy.

Molecular-behavioural relationships were explored *post hoc* using Spearman rank correlations (*ρ*) between group-level mean gene expression ([C]rel) and group-level mean behavioural indices across seven groups, comprising the intact Control group plus the six therapy groups (*n* = 7 group means per correlation). Spearman was chosen as a non-parametric measure appropriate for the small sample size and non-normal distributions; given the exploratory nature and limited power of group-level analyses, reported associations are uncorrected and should be treated as hypothesis-generating.

## Results

### Behavioural tests results

#### Elevated Plus Maze

**Figure 1:**
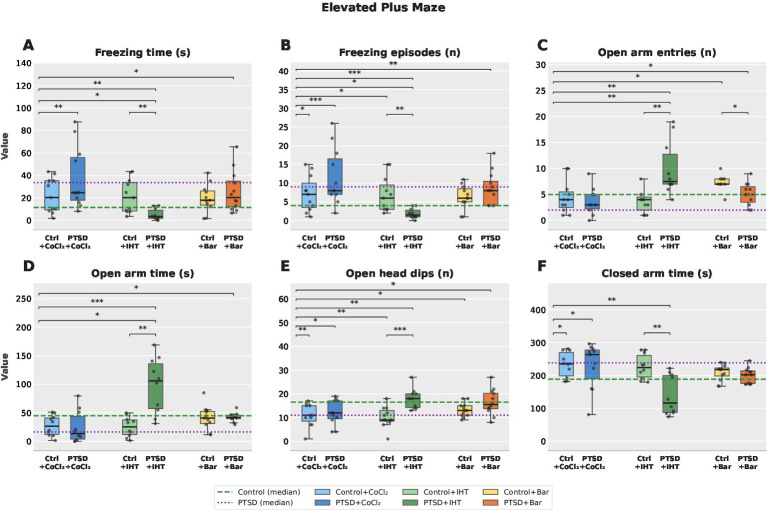
Elevated plus maze results. Box plots show freezing time **(A)**, freezing episodes **(B)**, open arm entries **(C)**, open arm time **(D)**, open head dips **(E)**, and closed arm time **(F)** across experimental groups: control+CoCl_2_, PTSD+CoCl_2_, control+IHT, PTSD+IHT, control+Bar, and PTSD+Bar. Green dashed and purple dotted horizontal lines indicate the median values for the intact control and PTSD groups, respectively. Individual data points represent single animals. Statistical analysis as described for figure. *p < 0.05, **p < 0.01, ***p < 0.001.

In the Elevated Plus Maze, open-arm time in control animals was 54.2 ± 42.2 s, whereas in PTSD animals it was ~52% lower (26.2 ± 31.7 s; *p* = 0.027). Intermittent hypoxia was the only method with a consistent anti-anxiety effect within the PTSD group. PTSD animals with this type of hypoxia showed a significant reduction in freezing time relative to Control+IHT animals (5.360 ± 4.934 s vs. 21.609 ± 14.925 s; *p* < 0.01) (Figure 1A), alongside fewer freezing episodes (1.900 ± 1.370 vs. 7.000 ± 4.775; *p* < 0.01) (Figure 1B). They also entered the open arms more frequently (9.900 ± 5.216 vs. 3.545 ± 2.115; *p* < 0.01) (Figure 1C) and spent markedly more time there (97.920 ± 48.259 s vs. 25,300 ± 17.428 s; *p* < 0.01) (Figure 1D). Open head dips—a measure of risk-assessment behaviour—were also higher in PTSD with intermittent hypoxia than in the control group with this type of treatment (18.000 ± 4.269 vs. 10.182 ± 4.446; *p* < 0.001) (Figure 1E), and closed arm time was reduced (139.660 ± 59.900 s vs. 226.870 ± 37.953 s; *p* = 0.008) (Figure 1F). Taken together, these results indicate that IHT substantially attenuates the hypervigilant, avoidance-dominated behavioural profile characteristic of the PTSD phenotype. Neither CoCl₂ nor Bar hypoxia produced significant within-PTSD improvements on EPM indices, suggesting that the anxiolytic action in this test is specific to the IHT protocol.

In non-stressed animals, both CoCl₂ and IHT reduced total locomotion compared to controls, with Control+CoCl₂ also increasing freezing episodes and closed arm time. Control+IHT increased freezing and decreased open-head dips and closed-arm entries. Rotations decreased in all control groups. Control+Bar animals showed a modest rise in open arm entries. These results indicate that, without prior stress, hypoxic treatments like CoCl₂ and IHT cause mild anxiety-like or hypolocomotive effects, contrasting with their effects in PTSD animals.

#### Open Field Test

**Figure 2:**
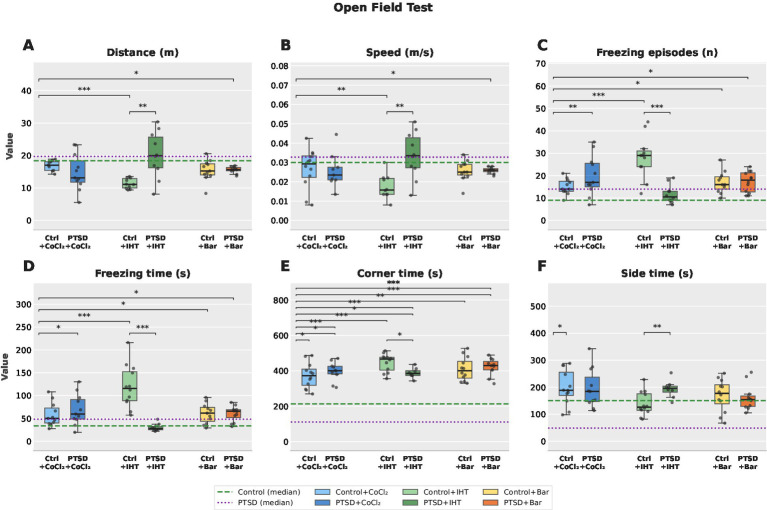
Open Field Test results. Boxplots show distance travelled **(A)**, locomotion speed **(B)**, freezing episodes **(C)**, freezing time **(D)**, corner time **(E)**, and side time **(F)** across experimental groups: Control+CoCl_2_, PTSD+CoCl_2_, Control+IHT, PTSD+IHT, Control+Bar, and PTSD+Bar. Green dashed and purple dotted horizontal lines indicate the median values for the intact Control and PTSD groups, respectively. Individual data points represent single animals. Outliers were removed using the IQR 1.5 × method. Normality was assessed using the Shapiro–Wilk test; group differences were evaluated using one-way ANOVA or the Kruskal–Wallis test, with post-hoc tests using the *t*-test or Mann–Whitney U test. Significance brackets denote pairwise comparisons: *p < 0.05, **p < 0.01, ***p < 0.001.

In the Open Field Test, no parameter differed significantly between groups (e.g., freezing time 42.0 ± 34.1 s in controls vs. 59.5 ± 42.0 s in PTSD, ~42% higher; *p* = 0.16). Intermittent hypoxia produced the clearest within-PTSD effect in the OFT. PTSD animals after this type of hypoxia travelled significantly greater distances than the control group with the same treatment (20.157 ± 7.146 m vs. 11.359 ± 1.582 m; *p* < 0.001) (Figure 2A). They also had higher mean speed (0.034 ± 0.012 m/s vs. 0.017 ± 0.006 m/s; *p* < 0.01) (Figure 2B), with substantially fewer freezing episodes (11.700 ± 4.084 vs. 28.182 ± 9.516; *p* < 0.001) (Figure 2C) and shorter total freezing time (30.250 ± 8.636 s vs. 119.900 ± 46.345 s; *p* < 0.001) (Figure 2D). Corner time was also lower in PTSD with IHT than in control with IHT (388.562 ± 27.847 s vs. 446.408 ± 50.844 s; *p* < 0.05) (Figure 2E), and side time was reduced (138.600 ± 44.322 s vs. 193.737 ± 32.695 s; *p* < 0.01) (Figure 2F). These results are consistent with the EPM data and reinforce the interpretation that IHT selectively normalises locomotor and anxiety-like behaviour in the context of prior traumatic stress. No significant within-PTSD improvements were observed for the CoCl₂ or Bar groups in the OFT.

Control animals treated with IHT showed increased anxiety-like behaviour in the OFT, travelling less, moving more slowly, freezing, entering the centre less, and spending more time in the corner compared to naive controls. Control animals with applied CoCl₂ also showed more corner entries and time. Both control and PTSD groups with Bar exhibited more freezing episodes and spent more time in the corner.

#### Dark–Light Box

**Figure 3:**
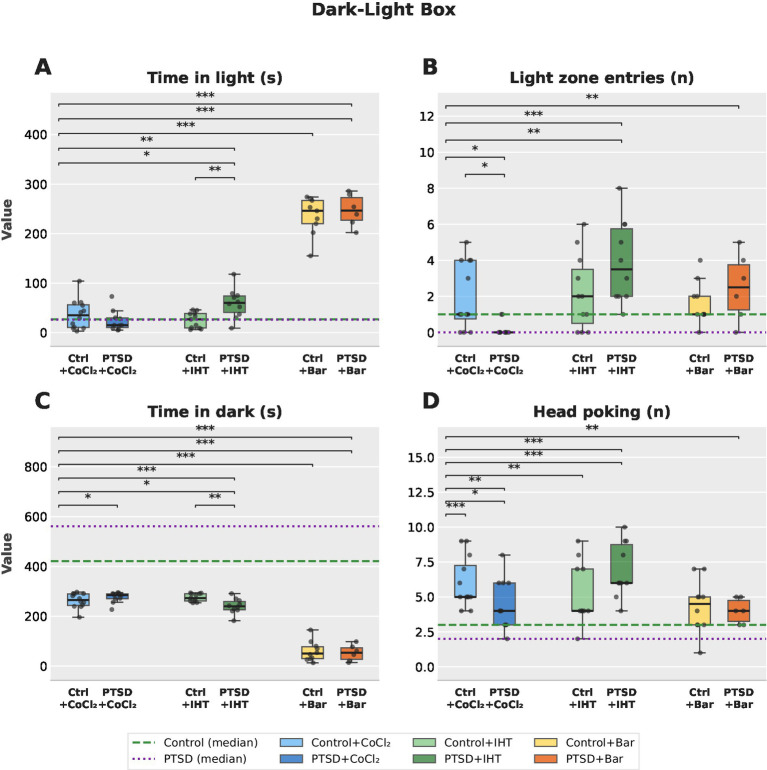
Dark–Light Box results. Boxplots show time in light zone **(A)**, light zone entries **(B)**, time in dark zone **(C)**, and head poking episodes **(D)** across experimental groups: Control+CoCl_2_, PTSD+CoCl_2_, Control+IHT, PTSD+IHT, Control+Bar, and PTSD+Bar. Green dashed and purple dotted horizontal lines indicate the median values for the intact Control and PTSD groups, respectively. Individual data points represent single animals. Statistical analysis as described for Figure 1. *p < 0.05, **p < 0.01, ***p < 0.001.

In the Dark–Light Box, control animals made 2.33 ± 1.52 light-zone entries, whereas PTSD animals entered the light zone ~44% less often (1.31 ± 0.60; *p* = 0.009). PTSD animals after intermittent hypoxia spent significantly more time in the light compartment than Control+IHT counterparts (59.100 ± 30.120 s vs. 25.000 ± 15.937 s; *p* < 0.01) (Figure 3A) and entered it more frequently than native control (4.900 ± 2.283 vs. 2.333 ± 1.523; *p* < 0.001) (Figure 3B). PTSD animals after barometric chamber treatment showed a remarkable increase in light zone time (247.167 ± 32.431 s; *p* < 0.001) compared to naive controls. This also corresponds with a reduction in dark zone time (52.833 ± 32.431 s vs. 403.625 ± 173.220 s; *p* < 0.001) (Figure 3C). CoCl₂-treated PTSD animals, in contrast, entered the light zone less frequently than naive controls (1.222 ± 0.441 vs. 2.333 ± 1.523; *p* < 0.05) and showed fewer entries than the control group with the same treatment (*p* < 0.05).

As for the Bar group, both the PTSD and control groups were marked by a preference for the light section and less time in the dark, indicating reduced anxiety-like behaviour. On the other hand, control and PTSD groups treated with intermittent hypoxia had increased exploratory movement, as they had more frequent transitions between compartments.

### Molecular analysis results

**Figure 4:**
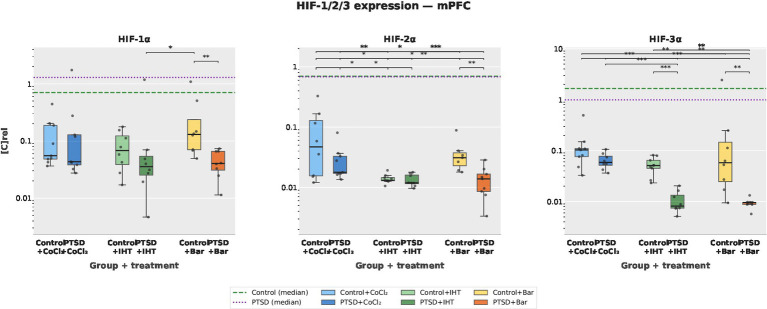
HIF isoforms expression in the cortex following hypoxic interventions in a rat PTSD model. Relative expression ([*C*]rel) of HIF-1α **(A)**, HIF-2α **(B)**, and HIF-3α **(C)** in the mPFC of rats subjected to SPS protocol and treated with CoCl_2_, IHT, or Bar. Green dashed line—Control baseline median; purple dotted line—PTSD baseline median. Boxplots represent median, interquartile range, and 1.5 × IQR whiskers; individual data points shown. Note the logarithmic scale. Significance brackets indicate pairwise comparisons between Control+therapy and PTSD+therapy groups after Benjamini-Hochberg FDR correction (per gene). *p < 0.05, **p < 0.01, ***p < 0.001.

**Figure 5:**
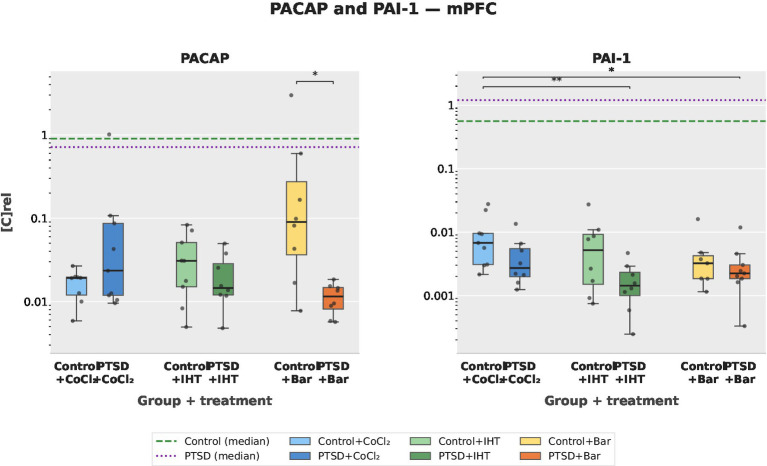
HIF isoforms expression in the hippocampus following hypoxic interventions in a rat PTSD model. Relative expression ([C]rel) of HIF-1α **(A)**, HIF-2α **(B)**, and HIF-3α **(C)** in the hippocampus of rats subjected to the Single Prolonged Stress (SPS) protocol and treated with cobalt chloride (CoCl₂), intermittent hypoxia training (IHT), or hypobaric chamber exposure (Bar). Green dashed line Control baseline median; purple dotted line PTSD baseline median. Boxplots represent median, interquartile range, and 1.5 × IQR whiskers; individual data points shown. Note the logarithmic scale. Significance brackets indicate pairwise comparisons between Control+therapy and PTSD+therapy groups after Benjamini-Hochberg FDR correction (per gene). *p < 0.05, **p < 0.01, ***p < 0.001.

In untreated PTSD animals, HIF-1α expression was significantly elevated in the hippocampus relative to controls (2.78 ± 1.97 vs. 1.10 ± 0.50; *q* = 0.039), and HIF-2α and HIF-3α showed a similar, non-significant upward trend in the same region (HIF-2α: 2.05 ± 1.33 vs. 1.24 ± 1.05; HIF-3α: 32.5 ± 44.7 vs. 6.7 ± 14.0; both ns), reflecting large between-animal variability. In the mPFC, none of the three HIF subunits differed significantly between PTSD and control animals, and for HIF-2α and HIF-3α the PTSD means were numerically lower, not higher, than control. Taken together, the hippocampus shows a more consistent hypoxia-related transcriptional response to trauma than the mPFC, but only HIF-1α reaches statistical significance at baseline. All three hypoxic interventions significantly reduced HIF-1α and HIF-2α mRNA in both regions, in both Control- and PTSD-background animals. HIF-3α showed a different pattern: suppression relative to baseline reached significance in PTSD-background animals under all three regimens in both regions, but was largely absent in Control-background animals (significant only for Control+IHT in the mPFC), indicating that the HIF-3α response to hypoxic conditioning is itself substantially trauma-dependent (Figures 4, 5). IHT produced the deepest overall suppression of HIF subunits; for HIF-3α this suppression was significantly greater in stressed than in non-stressed animals, whereas for HIF-1α and HIF-2α no significant Control vs. PTSD difference was observed under IHT. Bar suppressed all three HIF subunits significantly more strongly in stressed than in non-stressed animals, although residual HIF-1α expression in the Bar group remained higher than under IHT. CoCl₂ produced uniform, PTSD-independent suppression of HIF-1α and HIF-2α; its otherwise strong suppression of HIF-3α, like that of the other two regimens, reached significance only in PTSD-background animals, not in Control-background animals.

**Figure 6:**
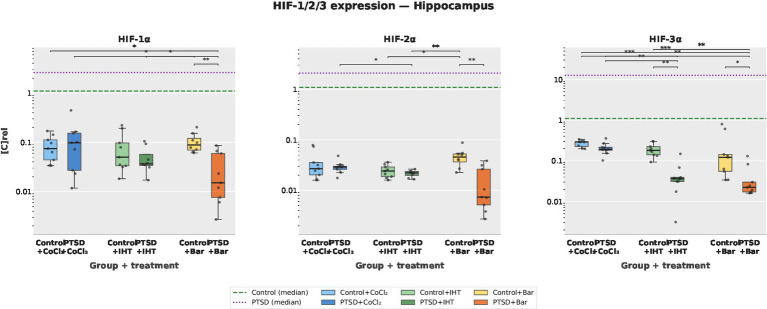
PACAP **(A)** and PAI-1 **(B)** expression in the cortex following hypoxic interventions in a rat PTSD model. Relative expression ([C]rel) of PACAP and PAI-1 in the mPFC of rats subjected to the SPS protocol and treated with CoCl_2_, IHT, or Bar. Green dashed line—Control baseline median; purple dotted line—PTSD baseline median. Boxplots represent median, interquartile range, and 1.5 × IQR whiskers; individual data points shown. Note the logarithmic scale. Significance brackets indicate pairwise comparisons between Control+therapy and PTSD+therapy groups after Benjamini-Hochberg FDR correction (per gene). *p < 0.05, **p < 0.01, ***p < 0.001.

**Figure 7:**
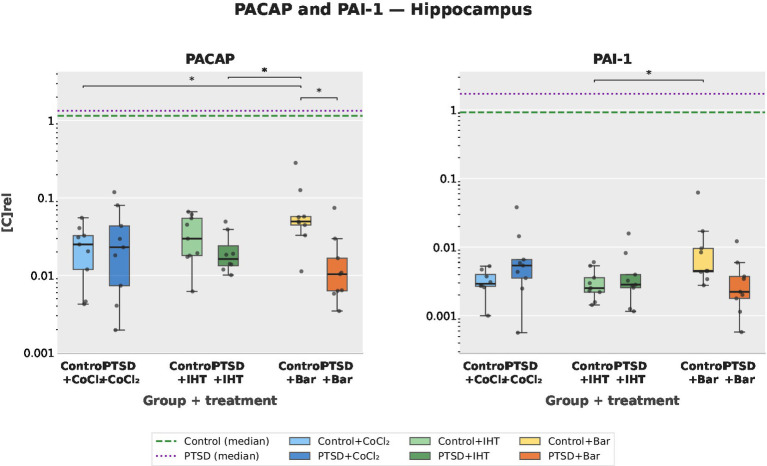
PACAP **(A)** and PAI-1 **(B)** expression in the hippocampus following hypoxic interventions in a rat PTSD model. Relative expression ([C]rel) of PACAP and PAI-1 in the hippocampus of rats subjected to the Single Prolonged Stress (SPS) protocol and treated with cobalt chloride (CoCl_2_), intermittent hypoxia training (IHT), or hypobaric chamber exposure (Bar). Green dashed line: Control baseline median; purple dotted line: PTSD baseline median. Boxplots represent median, interquartile range, and 1.5 × IQR whiskers; individual data points shown. Note the logarithmic scale. Significance brackets indicate pairwise comparisons between Control+therapy and PTSD+therapy groups after Benjamini-Hochberg FDR correction (per gene). *p < 0.05, **p < 0.01, ***p < 0.001.

PACAP and PAI-1 expression in untreated PTSD animals did not differ significantly from controls in either region, but both transcripts were markedly reduced by all hypoxic interventions (Figures 6, 7). PACAP suppression was numerically weakest under Bar in both regions, reaching significance in the hippocampus only relative to CoCl₂; PAI-1 suppression was significantly weaker under Bar than under IHT in the hippocampus, but not distinguishable across regimens in the mPFC. Bar was also the only regimen producing a significant within-therapy, stress-dependent effect on PACAP (suppression significantly stronger in PTSD+Bar than in Control+Bar in both regions). A comparable stress-dependence, however, was independently observed for HIF-1α and HIF-2α under Bar and, most strikingly, for HIF-3α under all three regimens (see below), indicating that stress-dependent hypoxic signalling is not confined to a single gene–regimen combination.

#### Medial prefrontal cortex

In mPFC, HIF-1α expression levels showed no significant difference in control vs. PTSD rats. All interventions significantly suppressed HIF-1α expression relative to both Control and PTSD baselines (*q* < 0.05 to < 0.001). Amongst PTSD-treated animals, cross-therapy comparisons revealed no significant differences between PTSD+IHT (0.178 ± 0.406), PTSD+CoCl₂ (0.272 ± 0.557), and PTSD+Bar (0.044 ± 0.022) (all ns), so no single modality could be identified as the most effective suppressor of HIF-1α in this tissue. A significant within-therapy PTSD vs. Control difference was observed only for Bar (PTSD+Bar 0.044 ± 0.022 vs. Control+Bar 0.274 ± 0.361, *q* = 0.008), whereas CoCl₂ and IHT suppressed HIF-1α similarly regardless of prior PTSD induction (ns).

Over the 14-day protocol, CoCl₂ acts by stabilising HIF-1α protein, which triggers negative feedback on HIF1A transcription; therefore, the “suppression” observed in qPCR reflects reduced mRNA abundance rather than reduced HIF-1α protein. In contrast, under Bar exposure, sustained PHD activity may permit adaptive adjustment of both transcript levels and protein stabilisation, so the mRNA signal has a different mechanistic meaning in CoCl₂- versus Bar-treated groups.

HIF-2α expression control vs. PTSD showed no significant difference in expression in mPFC. HIF-2α was uniformly and profoundly suppressed by IHT (Control+IHT 0.014 ± 0.002; PTSD+IHT 0.013 ± 0.003) relative to both baselines (*q* < 0.001), with no difference between Control+IHT and PTSD+IHT (ns). CoCl₂ similarly produced non-selective suppression (Control+CoCl₂ 0.093 ± 0.110; PTSD+CoCl₂ 0.029 ± 0.021). In contrast, Bar treatment showed a divergent pattern: Control+Bar retained markedly higher HIF-2α expression (0.037 ± 0.024) than Control+IHT (*q* = 0.002), though not significantly different from Control+CoCl₂ (ns). At the same time, PTSD+Bar (0.014 ± 0.008) was significantly lower than Control+Bar (*q* = 0.007), suggesting that prior PTSD sensitises the mPFC HIF-2α response to this hypoxic regimen.

HIF-3α expression control vs. PTSD showed no significant difference in expression in mPFC. For HIF-3α, CoCl₂ significantly suppressed expression in PTSD-background animals relative to their own PTSD baseline (*q* < 0.001) but produced no significant within-therapy PTSD vs. Control difference (ns). Both IHT and Bar produced significant PTSD-selective suppression: PTSD+IHT (0.011 ± 0.005) was significantly lower than Control+IHT (0.052 ± 0.021, *q* < 0.001), and PTSD+Bar (0.009 ± 0.002) was significantly lower than Control+Bar (0.371 ± 0.840, *q* = 0.004), indicating that the PTSD-selective HIF-3α response in the mPFC is not unique to IHT but is shared with Bar.

PACAP expression control vs. PTSD showed no significant difference in expression in mPFC. PACAP was significantly suppressed by all three interventions relative to their respective baselines (*q* < 0.05 to < 0.001). Cross-therapy comparisons showed that Control+Bar (0.501 ± 1.026) retained significantly higher PACAP than PTSD+Bar (0.011 ± 0.005, *q* = 0.029); Control+Bar was numerically, but not significantly, higher than Control+IHT (0.035 ± 0.028) or Control+CoCl₂ (0.017 ± 0.007) (both ns). The within-therapy elevation of PACAP retained in Control+Bar but lost in PTSD+Bar animals remains the clearest dissociation amongst the markers examined, although it is now supported by the within-therapy comparison rather than by cross-therapy significance.

PAI-1 expression control vs. PTSD showed no significant difference in expression in mPFC. PAI-1 was consistently and significantly suppressed by all interventions relative to baseline (*q* < 0.001), with no significant within-therapy PTSD vs. Control differences for any treatment in the mPFC, indicating PTSD-independent suppression of PAI-1 by all three modalities. In the recalculated dataset, cross-therapy comparisons showed no significant differences in mPFC PAI-1 levels amongst Control+Bar (0.005 ± 0.005), Control+IHT (0.008 ± 0.009), and Control+CoCl₂ (0.010 ± 0.009) (all ns).

#### Hippocampus

In hippocampus, PTSD animals showed significantly increased HIF-1α expression relative to control (2.783 ± 1.966 vs. 1.100 ± 0.498; *q* = 0.039). HIF-1α expression was suppressed by all three interventions relative to both baselines. Cross-therapy comparisons revealed no significant differences amongst PTSD+IHT (0.050 ± 0.032), PTSD+CoCl₂ (0.122 ± 0.133), and PTSD+Bar (0.031 ± 0.031) (all ns). A significant within-therapy PTSD vs. Control difference was observed only for Bar (PTSD+Bar 0.031 ± 0.031 vs. Control+Bar 0.105 ± 0.050, *q* = 0.006); neither IHT nor CoCl₂ showed a significant within-therapy effect in this tissue (ns), indicating that PTSD-selective suppression of hippocampal HIF-1α is specific to Bar rather than IHT.

HIF-2α expression control vs. PTSD showed no significant difference in hippocampal expression. Suppression by CoCl₂ and IHT was non-selective (no within-therapy PTSD vs. Control differences, ns). As observed in the mPFC, the barochamber group showed a PTSD-dependent pattern: PTSD+Bar (0.015 ± 0.014) was significantly lower than Control+Bar (0.047 ± 0.020, *q* = 0.004), whilst Control+Bar itself retained significantly higher expression than Control+IHT (0.025 ± 0.007, *q* = 0.011), though not significantly higher than Control+CoCl₂ (0.036 ± 0.024, ns). This PTSD-dependent Bar pattern was broadly consistent across both regions, although the cross-therapy elevation of Control+Bar over Control+CoCl₂ that was significant in the mPFC did not reach significance in the hippocampus.

HIF-3α expression control vs. PTSD showed no significant difference in hippocampal expression. For HIF-3α, the hippocampus showed a broad profile of PTSD-specific effects, similar to the mPFC. Both IHT and barochamber produced significant within-therapy differences: PTSD+IHT (0.046 ± 0.042) was significantly lower than Control+IHT (0.193 ± 0.072, *q* = 0.003), and PTSD+Bar (0.040 ± 0.040) was significantly lower than Control+Bar (0.222 ± 0.282, *q* = 0.013). CoCl₂ again showed no within-therapy difference (ns). Cross-therapy comparisons showed that PTSD+IHT was significantly lower than PTSD+CoCl₂ (0.207 ± 0.072, *q* = 0.002) but not significantly different from PTSD+Bar (ns), indicating that IHT and Bar were comparably effective PTSD-selective suppressors of hippocampal HIF-3α, both outperforming CoCl₂.

PACAP expression control vs. PTSD showed no significant difference in hippocampal expression. PACAP in the hippocampus again showed the clearest region-specific dissociation. Control+Bar retained elevated expression (0.079 ± 0.083) that was significantly higher than Control+CoCl₂ (0.025 ± 0.017, *q* = 0.030), though not significantly different from Control+IHT (0.035 ± 0.022, ns). PTSD+Bar (0.018 ± 0.023) was significantly lower than Control+Bar (*q* = 0.015) and did not differ from PTSD+IHT or PTSD+CoCl₂ (ns). This within-therapy dissociation between Control+Bar and PTSD+Bar for hippocampal PACAP suggests that prior PTSD alters the hippocampal neuropeptide response to barochamber-induced hypoxia, although the cross-therapy elevation of Control+Bar is now only partially supported (significant vs. CoCl₂ but not vs. IHT). IHT and CoCl₂ produced uniform, PTSD-independent PACAP suppression.

PAI-1 expression control vs. PTSD showed no significant difference in hippocampal expression. PAI-1 in the hippocampus was significantly suppressed by all interventions relative to baseline (*q* < 0.001). No within-therapy PTSD vs. Control differences were observed for any of the three regimens (ns). Cross-therapy analysis showed that Control+IHT (0.003 ± 0.002) was significantly lower than Control+Bar (0.013 ± 0.019, *q* = 0.043), but not significantly different from Control+CoCl₂ (0.003 ± 0.001, ns); PTSD+IHT (0.005 ± 0.005) and PTSD+Bar (0.004 ± 0.004) did not differ significantly (ns). The previously reported IHT advantage in hippocampal PAI-1 suppression is therefore only partially supported in the recalculated dataset, limited to the Control-background comparison with Bar.

Across both brain regions, IHT and Bar showed comparably deep, significant PTSD-selective suppression of HIF-3α, whereas a significant PTSD-selective effect for HIF-1α was observed only for Bar, and only in the hippocampus. Barochamber treatment in fact showed the broadest pattern of PTSD-selective within-therapy effects overall, spanning HIF-1α (hippocampus), HIF-2α (both regions), HIF-3α (both regions), and PACAP (both regions). CoCl₂ suppressed all markers relative to baseline but without significant within-therapy PTSD specificity for any gene in either region. Cross-therapy differences in PAI-1 suppression, previously attributed mainly to IHT, were substantially attenuated in the recalculated dataset. These results indicate that, at the transcriptional level, Bar shows the most consistently PTSD-selective molecular signature amongst the three regimens, even though only IHT produced a PTSD-selective behavioural benefit—a dissociation between molecular and behavioural PTSD-selectivity that is addressed further in the Discussion.

#### Correlation analysis: gene expression and behavioural outcomes

**Figure 8:**
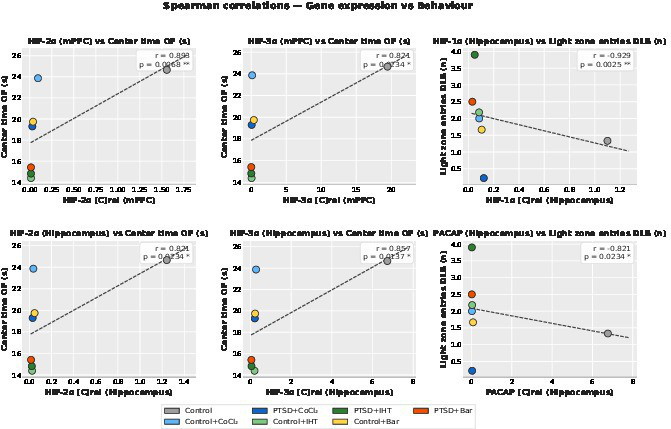
Spearman correlations between group-mean gene expression ([C]rel, x-axis) and group-mean behavioural indices (y-axis) across seven groups (intact Control plus six therapy groups). Each point represents one group mean (circles—Control background, squares—PTSD background; colour: Blue—CoCl_2_, green—IHT, yellow/orange—barochamber). Dashed lines indicate linear regression trends (for visual guidance only). Insets report Spearman *ρ* and uncorrected *p*-value. All significant correlations are shown (*p* < 0.05, uncorrected); in the recalculated dataset these involve only Open Field centre time and Dark–Light Box light-zone entries. OF, Open Field; DL, Dark–Light Box.

To explore the relationship between molecular and behavioural changes induced by hypoxic therapies, we performed Spearman rank correlations between group-level mean expression values and behavioural indices (methodology detailed in Statistical Analysis). Of 80 tested pairs, 6 reached significance (*p* < 0.05) after unadjusted comparison; these are reported below and illustrated in Figure 8.

In the recalculated dataset, the pattern of significant correlations is markedly different from the earlier analysis and is dominated by two behavioural parameters rather than by gene-specific relationships. Center time in the Open Field correlated positively with cortical HIF-2α (*ρ* = 0.893, *p* = 0.007) and cortical HIF-3α (*ρ* = 0.821, *p* = 0.023): groups with deeper suppression of these transcripts spent less time in the arena centre. None of the correlations reported in the earlier, uncorrected analysis (cortical HIF-1α or PACAP with EPM closed-arm time or OF freezing) remained significant.

In the hippocampus, Center time in the Open Field again correlated positively with HIF-2α (*ρ* = 0.821, *p* = 0.023) and HIF-3α (*ρ* = 0.857, *p* = 0.014), mirroring the cortical pattern. Light-zone entries in the Dark–Light Box correlated negatively with hippocampal HIF-1α (*ρ* = −0.929, *p* = 0.003) and hippocampal PACAP (*ρ* = −0.821, *p* = 0.023): groups with deeper suppression of these two transcripts made more entries into the illuminated compartment. The near-identical correlation coefficients across different genes for the same behavioural parameter, in both regions, suggest that these associations track the overall depth of hypoxic suppression across the transcriptional programme—which co-varies tightly across HIF-2α, HIF-3α, and, for the Dark–Light Box measure, HIF-1α and PACAP—rather than reflecting a gene-specific molecular–behavioural relationship. None of the correlations reported in the earlier, uncorrected analysis (hippocampal PACAP with OF locomotion, hippocampal HIF-1α or HIF-3α with EPM indices, or cortical PAI-1 with DLB head-poking) remained significant in the recalculated dataset.

Taken together, these correlations indicate that, at the group level, greater exploration of the open-field centre and greater light-zone entry co-occur with deeper suppression of HIF-2α and HIF-3α (and, for light-zone entries, HIF-1α and PACAP) across both brain regions, consistent with a general link between hypoxia-related transcriptional suppression and reduced anxiety-like behaviour. However, the near-identical coefficients across genes for a given behavioural parameter indicate substantial collinearity amongst the transcripts, so these results are better read as evidence of a shared suppression axis than as gene-specific molecular-behavioural links. As before, these findings should be interpreted with caution given the small number of group means per correlation; individual-level replication would be required to confirm any of these associations (Figure 8).

## Discussion

This study compared three hypoxic regimens—intermittent normobaric hypoxia (IHT), hypobaric hypoxia (Bar), or chemical hypoxia by cobalt chloride (CoCl₂),—in a rat single-prolonged-stress (SPS) model of PTSD, and assessed their behavioural effects together with the expression of HIF-1α, HIF-2α, HIF-3α, PACAP, and PAI-1 in the medial prefrontal cortex (mPFC) and hippocampus. The principal finding is that the three regimens produced markedly divergent behavioural outcomes despite a partly overlapping transcriptional signature, and that only IHT normalised both the behavioural and the molecular phenotype of traumatied animals.

The SPS model is well validated for the induction of an anxiety-like phenotype, with reliable changes in EPM and DLB indices but variable effects in the OFT (Liberzon et al., 1997; Lisieski et al., 2018; Chaby et al., 2019; Nahvi et al., 2019), and our untreated PTSD animals reproduced this profile. Against this baseline, CoCl₂ failed to improve PTSD-related behaviour in EPM or OFT and worsened DLB performance through reduced light-zone entries. Bar produced a mixed profile: it did not improve EPM or OFT but markedly increased light-zone occupancy in the DLB even in non-stressed controls, a pattern consistent with sensorimotor side-effects of the chamber exposure rather than with genuine anxiolysis, given the well-documented sensitivity of the DLB to locomotor and exploratory state (Bourin and Hascoët, 2003). It should be noted, however, that general locomotor activity was not separately quantified in the OFT for the Bar groups, so a locomotor contribution to the increased DLB light-zone occupancy cannot be formally excluded and remains a limitation of the present interpretation. Only IHT reduced both EPM and OFT freezing in PTSD animals to levels indistinguishable from controls and increased open-arm exploration. This pattern—robust anxiolysis with IHT, weaker or adverse effects with CoCl₂ and Bar—is consistent with the framing of intermittent hypoxic conditioning as a stress-protective intervention whose efficacy depends on the cyclic structure, dose, and intensity of the hypoxic stimulus (Burtscher et al., 2024; Serebrovskaya and Xi, 2016; Navarrete-Opazo and Mitchell, 2014), and with the general adaptation framework introduced by Selye, in which beneficial outcomes require a stimulus pattern that engages adaptive rather than purely stressor responses (Selye, 1950).

The molecular data showed a region-specific pattern of basal HIF activation in untreated PTSD animals. HIF-1α was significantly elevated in the hippocampus relative to controls (2.78 ± 1.97 vs. 1.10 ± 0.50; *q* = 0.039), whilst HIF-2α and HIF-3α showed a similar, non-significant upward trend in the same region; none of the three subunits differed significantly between PTSD and control animals in the mPFC. This pattern—a confirmed, if modest, hippocampal HIF-1α elevation, with parallel but statistically inconclusive trends for HIF-2α and HIF-3α—is considerably less dramatic than an earlier, uncorrected estimate had suggested for HIF-2α specifically: large between-animal variability had inflated that comparison to an apparent ~50-fold difference, whereas the Grubbs-cleaned, recalculated dataset places it at approximately 1.7-fold and non-significant. We interpret the confirmed HIF-1α elevation as evidence of a modest, hippocampally-restricted hypoxia-related transcriptional response to chronic traumatic stress, rather than the more dramatic, HIF-2α-dominant pseudo-hypoxic signature previously proposed. Because HIF-1α has been more extensively studied in models of psychoemotional stress (Rybnikova et al., 2015; Burtscher et al., 2021), and because HIF-2α’s distinct roles in erythropoiesis, antioxidant defence, and vascular remodelling (Scortegagna et al., 2003; Rankin et al., 2007; Tian et al., 1997, 1998) are not strongly implicated by a non-significant trend, HIF-1α rather than HIF-2α is the more defensible candidate for a trauma-associated hippocampal hypoxia signal in this dataset. All three interventions reduced hippocampal HIF-2α to a similarly low residual level relative to untreated PTSD (PTSD+CoCl₂ 0.029 ± 0.008; PTSD+IHT 0.022 ± 0.003; PTSD+Bar 0.015 ± 0.014; all ns relative to one another), with no significant modality-dependent difference in residual HIF-2α tone—in contrast to an earlier suggestion that Bar left more residual HIF-2α than the other regimens.

HIF-1α was suppressed by all three regimens in both regions, without a consistent, statistically significant ranking amongst modalities. That a hypoxic stimulus should reduce HIF-1α mRNA may appear counter-intuitive, but HIF-1α mRNA is not directly oxygen-regulated at the transcriptional level (Huang and Bunn, 2003), and chronic or conditioned hypoxic exposure can suppress HIF-1α through negative feedback involving HIF-3α and other regulators (Duan, 2015). Across our 14-day protocol, the apparent suppression therefore reflects an adaptive set-point: under IHT and Bar, HIF-1α protein has been transiently stabilised and the system has down-regulated its own transcript pool by feedback; under CoCl₂, the persistently stabilised HIF-1α protein triggers feedback inhibition of HIF1A transcription (Stiehl et al., 2006; Dai et al., 2014). A low HIF1A mRNA signal under CoCl₂ therefore does not imply a small functional HIF response, and may indeed indicate the opposite. In the recalculated dataset, the negative correlation between hippocampal HIF-1α expression and Dark–Light Box light-zone entries (*ρ* = −0.929) suggests that groups with less-suppressed HIF-1α made fewer entries into the illuminated compartment, consistent with a link between residual HIF-1α tone and reduced light-seeking exploration, although the direction of this association cannot be inferred from the present data.

HIF-3α showed the most consistent PTSD-selective effect of any marker. In the recalculated dataset, both IHT and Bar produced significant within-therapy PTSD vs. Control suppression of HIF-3α in both the mPFC and the hippocampus—never CoCl₂. Because HIF-3α is partly a negative regulator of HIF-1α and HIF-2α (Makino et al., 2001), its preferential suppression in traumatised animals under IHT and Bar may reflect altered feedback regulation of the HIF system after cyclic and sustained hypoxic exposure, respectively. The absence of this within-therapy effect under CoCl₂ may indicate that the systemic and ventilatory components recruited by inhalation-based hypoxic exposures contribute to HIF-3α dynamics in the PTSD brain in ways that direct PHD inhibition does not (Prabhakar, 2016; Prabhakar et al., 2020; Portnychenko et al., 2013).

PACAP expression was reduced by all three interventions in both regions, with one notable exception: Control+Bar animals retained markedly elevated PACAP, particularly in the hippocampus, whilst PTSD+Bar animals showed suppression to the level of the other treated groups. This dissociation was not observed for IHT or CoCl₂, both of which suppressed PACAP uniformly regardless of trauma status. PACAP is a neuropeptide implicated in stress, fear, and trauma (Ressler et al., 2011; Stroth et al., 2011; Hashimoto et al., 2011; Velasco et al., 2022; Riser and Norrholm, 2022), and the trauma-dependent failure to retain Bar-induced PACAP elevation in the hippocampus suggests that prior PTSD reshapes the hippocampal neuropeptide response to barochamber hypoxia. The negative correlation between hippocampal PACAP and Dark–Light Box light-zone entries at the group level is consistent with the broader literature linking sustained PACAP elevation to passive, fear-driven behavioural states, although—as for HIF-1α, HIF-2α, and HIF-3α—this association is confounded by the collinearity of gene suppression across the transcriptional programme and cannot be attributed to PACAP specifically.

PAI-1 was consistently and significantly suppressed by all three interventions in both regions, with no PTSD-selective within-therapy difference. The direction of this effect is consistent with a neuroprotective interpretation, since reduced PAI-1 should permit greater tPA-mediated proBDNF cleavage and downstream BDNF signalling (Party et al., 2019). However, this uniform suppression was not accompanied by behavioural improvement in CoCl₂ or Bar animals, indicating that PAI-1 reduction alone is not a determinant of behavioural recovery in this model and that its functional consequences depend on the broader molecular context established by each intervention.

Two gradients emerge from the combined data, and their relationship is less straightforward than an earlier reading of the data suggested. The first is the depth of suppression of HIF-1α and PAI-1, which in the recalculated dataset does not scale consistently across regimens: cross-therapy differences amongst IHT, CoCl₂, and Bar were largely non-significant for both markers, in contrast to an earlier impression of a clear IHT-dominant gradient. The second is the presence of a PTSD-selective component, essentially absent under CoCl₂ but, contrary to our earlier reading, now more extensive under Bar (HIF-1α in the hippocampus, HIF-2α in both regions, HIF-3α in both regions, and PACAP in both regions) than under IHT (HIF-3α in both regions only). The behavioural data do not track this molecular gradient: only IHT, not Bar, produced consistent behavioural improvement, despite Bar showing the broader pattern of PTSD-selective molecular change. This dissociation indicates that the therapeutic effect of hypoxic conditioning in this model does not simply track the breadth of PTSD-selective HIF-pathway suppression; some property specific to IHT—plausibly its cyclic, repeated-exposure structure, as opposed to the single sustained daily exposure used for Bar—appears necessary to translate a PTSD-selective molecular signature into behavioural benefit—properties of dose, intensity, and temporal structure that differ across the three regimens compared here (Burtscher et al., 2024; Navarrete-Opazo and Mitchell, 2014; Serebrovskaya et al., 2013).

The exploratory Spearman correlations between group-mean expression and behavioural indices offer a different kind of support for this reading. Rather than pointing to specific genes, the recalculated correlations are dominated by two behavioural parameters—Open Field centre time and Dark–Light Box light-zone entries—each correlating almost identically with several HIF-pathway transcripts in both regions. This pattern is consistent with a shared suppression axis across the transcriptional programme rather than with gene-specific molecular-behavioural links, and it no longer implicates cortical HIF-1α, cortical PACAP, or hippocampal PAI-1, which drove the associations in an earlier, uncorrected analysis. These correlations must, in any case, be treated as hypothesis-generating rather than confirmatory: they are based on seven group-level means and are sensitive to extreme groups. Individual-level replication in a larger sample would be required before drawing mechanistic conclusions. Future studies designed with larger group sizes and individual-animal sampling could examine these molecular–behavioural relationships in greater detail, for example by correlating per-animal expression with behavioural indices, by testing the directionality of the associations identified here, and by determining whether the collinearity amongst HIF-pathway transcripts observed here reflects a common upstream regulator or simply the shared magnitude of hypoxic suppression across genes.

Two practical implications follow. First, IHT’s anxiolytic effect was state-dependent: beneficial in traumatised animals and mildly anxiogenic in non-stressed controls, consistent with a conditioning mechanism preferentially recruited in the traumatised brain. Such state-dependent benefit is a desirable property of a non-pharmacological intervention, since it would naturally limit unwanted effects in non-indicated populations. Second, the trauma-dependent failure of the hippocampal PACAP response under Bar indicates that prior PTSD reshapes how the brain responds to hypoxic conditioning, which has direct implications for personalising hypoxia-based interventions. Together, these observations support the cautious development of cyclic, normobaric IHT as a candidate non-pharmacological modulator of trauma-related disorders, whilst emphasising that intensity, modality, and dosing remain critical determinants of outcome.

dose and exposure duration; the distinct molecular and behavioural profile of CoCl₂ animals may therefore reflect either the chemical modality, the shorter exposure window, or a combination of both. Third, the design did not formally power individual-level molecular–behavioural correlation analysis; the Spearman associations reported here are based on seven group means, are affected by collinearity amongst the measured transcripts, and are hypothesis-generating rather than confirmatory. These limitations bound the strength of any modality-specific causal claim but do not undermine the principal observation that, across regimens equated for the intent to induce hypoxic adaptation, only mild intermittent normobaric hypoxia consistently normalised both behavioural indices and the hypoxia-related transcriptional readout in trauma-exposed animals.

## Data Availability

The datasets presented in this study can be found in online repositories. The names of the repository/repositories and accession number(s) can be found in the article/Supplementary material.
