# Supplementary material for: Mode-dependent effects of chemical, intermittent, and hypobaric hypoxia on HIF-1α, HIF-2α, HIF-3α, PACAP, and PAI mRNA expression in the single prolonged stress model of PTSD
Source: Front Neurosci. 2026 Jul 31;20:1904848. doi: 10.3389/fnins.2026.1904848 (PMC13473047; doi:10.3389/fnins.2026.1904848)
Supplement: Supplementary file 1 [file Table_1.DOCX]

**Supplementary Materials**

These supplementary tables accompany the manuscript “Mode-Dependent Effects of Chemical, Intermittent, and Hypobaric Hypoxia on HIF-1α, HIF-2α, HIF-3α, PACAP, and PAI mRNA Expression in the Single Prolonged Stress Model of PTSD.” Behavioural tables report all pairwise group comparisons that reached statistical significance (p < 0.05). qPCR tables report all 25 a priori pairwise comparisons per gene, with significance after Benjamini-Hochberg FDR correction performed independently per gene.

**Supplementary Table S1. Elevated Plus Maze (EPM) — all significant pairwise group comparisons.**

*Statistical workflow: Shapiro-Wilk normality test per group → one-way ANOVA (parametric) or Kruskal-Wallis (non-parametric) overall test → t-test or Mann-Whitney U for post-hoc pairwise comparisons. *p<0.05, **p<0.01, ***p<0.001.*

| **Parameter** | **Comparison** | **Overall test** | **p (overall)** | **Post-hoc** | **p (post-hoc)** | **Sig** | **Group 1: M±SD (n)** | **Group 2: M±SD (n)** |
| --- | --- | --- | --- | --- | --- | --- | --- | --- |
| Distance (m) | PTSD+CoCl₂ vs Control | ANOVA | <0.001 | t-test | 0.002 | ****** | 0.897 ± 0.322 (23) | 0.533 ± 0.219 (11) |
| Distance (m) | Ctrl+CoCl₂ vs Control | ANOVA | <0.001 | t-test | 0.001 | ****** | 0.897 ± 0.322 (23) | 0.523 ± 0.231 (12) |
| Distance (m) | PTSD+IHT vs Ctrl+IHT | ANOVA | <0.001 | t-test | 0.011 | ***** | 0.529 ± 0.233 (12) | 0.773 ± 0.155 (10) |
| Distance (m) | Ctrl+IHT vs Control | ANOVA | <0.001 | t-test | 0.001 | ****** | 0.897 ± 0.322 (23) | 0.529 ± 0.233 (12) |
| Freezing time (s) | PTSD+CoCl₂ vs Control | Kruskal | 0.001 | MWU | 0.004 | ****** | 12.809 ± 8.494 (23) | 37.291 ± 27.672 (11) |
| Freezing time (s) | PTSD+IHT vs Control | Kruskal | 0.001 | MWU | 0.028 | ***** | 12.809 ± 8.494 (23) | 5.360 ± 4.934 (10) |
| Freezing time (s) | PTSD+IHT vs Ctrl+IHT | Kruskal | 0.001 | MWU | 0.008 | ****** | 21.609 ± 14.925 (11) | 5.360 ± 4.934 (10) |
| Freezing time (s) | PTSD+Bar vs Control | Kruskal | 0.001 | MWU | 0.039 | ***** | 12.809 ± 8.494 (23) | 25.875 ± 18.052 (12) |
| Freezing episodes (n) | PTSD+CoCl₂ vs Control | ANOVA | <0.001 | t-test | <0.001 | ******* | 4.174 ± 2.902 (23) | 11.636 ± 7.580 (11) |
| Freezing episodes (n) | Ctrl+CoCl₂ vs Control | ANOVA | <0.001 | t-test | 0.030 | ***** | 4.174 ± 2.902 (23) | 7.182 ± 4.792 (11) |
| Freezing episodes (n) | PTSD+IHT vs Control | ANOVA | <0.001 | t-test | 0.025 | ***** | 4.174 ± 2.902 (23) | 1.900 ± 1.370 (10) |
| Freezing episodes (n) | Ctrl+IHT vs Control | ANOVA | <0.001 | t-test | 0.040 | ***** | 4.174 ± 2.902 (23) | 7.000 ± 4.775 (11) |
| Freezing episodes (n) | PTSD+IHT vs Ctrl+IHT | ANOVA | <0.001 | t-test | 0.004 | ****** | 7.000 ± 4.775 (11) | 1.900 ± 1.370 (10) |
| Freezing episodes (n) | PTSD+Bar vs Control | ANOVA | <0.001 | t-test | 0.002 | ****** | 4.174 ± 2.902 (23) | 8.500 ± 4.462 (12) |
| Open arm entries (n) | PTSD+IHT vs Control | Kruskal | 0.001 | MWU | 0.010 | ****** | 5.087 ± 2.729 (23) | 9.900 ± 5.216 (10) |
| Open arm entries (n) | PTSD+IHT vs Ctrl+IHT | Kruskal | 0.001 | MWU | 0.001 | ****** | 3.545 ± 2.115 (11) | 9.900 ± 5.216 (10) |
| Open arm entries (n) | Ctrl+Bar vs Control | Kruskal | 0.001 | MWU | 0.038 | ***** | 5.087 ± 2.729 (23) | 7.333 ± 1.581 (9) |
| Open arm entries (n) | PTSD+Bar vs Ctrl+Bar | Kruskal | 0.001 | MWU | 0.019 | ***** | 7.333 ± 1.581 (9) | 5.091 ± 2.212 (11) |
| Open arm time (s) | PTSD+IHT vs Control | Kruskal | 0.002 | MWU | 0.022 | ***** | 54.174 ± 42.196 (23) | 97.920 ± 48.259 (10) |
| Open arm time (s) | PTSD+IHT vs Ctrl+IHT | Kruskal | 0.002 | MWU | 0.001 | ****** | 25.300 ± 17.428 (10) | 97.920 ± 48.259 (10) |
| Open head dips (n) | PTSD+CoCl₂ vs Control | ANOVA | <0.001 | t-test | 0.015 | ***** | 19.917 ± 9.362 (24) | 12.091 ± 5.262 (11) |
| Open head dips (n) | Ctrl+CoCl₂ vs Control | ANOVA | <0.001 | t-test | 0.005 | ****** | 19.917 ± 9.362 (24) | 10.909 ± 4.763 (11) |
| Open head dips (n) | Ctrl+IHT vs Control | ANOVA | <0.001 | t-test | 0.003 | ****** | 19.917 ± 9.362 (24) | 10.182 ± 4.446 (11) |
| Open head dips (n) | PTSD+IHT vs Ctrl+IHT | ANOVA | <0.001 | t-test | 0.001 | ******* | 10.182 ± 4.446 (11) | 18.000 ± 4.269 (10) |
| Open head dips (n) | Ctrl+Bar vs Control | ANOVA | <0.001 | t-test | 0.022 | ***** | 19.917 ± 9.362 (24) | 13.250 ± 2.896 (12) |
| Closed arm entries (n) | Ctrl+CoCl₂ vs Control | ANOVA | 0.007 | t-test | 0.039 | ***** | 10.783 ± 5.300 (23) | 6.917 ± 4.502 (12) |
| Closed arm entries (n) | Ctrl+IHT vs Control | ANOVA | 0.007 | t-test | 0.031 | ***** | 10.783 ± 5.300 (23) | 6.545 ± 4.719 (11) |
| Closed arm entries (n) | PTSD+IHT vs Ctrl+IHT | ANOVA | 0.007 | t-test | 0.002 | ****** | 6.545 ± 4.719 (11) | 12.800 ± 3.011 (10) |
| Closed arm time (s) | PTSD+CoCl₂ vs Control | Kruskal | 0.004 | MWU | 0.043 | ***** | 187.483 ± 59.147 (23) | 229.982 ± 68.849 (11) |
| Closed arm time (s) | Ctrl+CoCl₂ vs Control | Kruskal | 0.004 | MWU | 0.033 | ***** | 187.483 ± 59.147 (23) | 233.180 ± 38.724 (10) |
| Closed arm time (s) | PTSD+IHT vs Ctrl+IHT | Kruskal | 0.004 | MWU | 0.008 | ****** | 226.870 ± 37.953 (10) | 139.660 ± 59.900 (10) |
| Rotations (n) | PTSD+CoCl₂ vs Control | Kruskal | <0.001 | MWU | 0.013 | ***** | 6.083 ± 1.586 (24) | 4.500 ± 1.354 (10) |
| Rotations (n) | Ctrl+CoCl₂ vs Control | Kruskal | <0.001 | MWU | 0.003 | ****** | 6.083 ± 1.586 (24) | 4.200 ± 0.789 (10) |
| Rotations (n) | Ctrl+IHT vs Control | Kruskal | <0.001 | MWU | 0.015 | ***** | 6.083 ± 1.586 (24) | 4.333 ± 2.015 (12) |
| Rotations (n) | PTSD+IHT vs Ctrl+IHT | Kruskal | <0.001 | MWU | 0.016 | ***** | 4.333 ± 2.015 (12) | 6.333 ± 0.866 (9) |
| Rotations (n) | PTSD+Bar vs Control | Kruskal | <0.001 | MWU | 0.007 | ****** | 6.083 ± 1.586 (24) | 4.455 ± 1.214 (11) |

**Supplementary Table S2. Open Field Test (OFT) — all significant pairwise group comparisons.**

*Same statistical workflow as Table S1.*

| **Parameter** | **Comparison** | **Overall test** | **p (overall)** | **Post-hoc** | **p (post-hoc)** | **Sig** | **Group 1: M±SD (n)** | **Group 2: M±SD (n)** |
| --- | --- | --- | --- | --- | --- | --- | --- | --- |
| Distance (m) | Ctrl+IHT vs Control | ANOVA | <0.001 | t-test | <0.001 | ******* | 17.909 ± 3.471 (21) | 11.359 ± 1.582 (10) |
| Distance (m) | PTSD+IHT vs Ctrl+IHT | ANOVA | <0.001 | t-test | 0.001 | ****** | 11.359 ± 1.582 (10) | 20.157 ± 7.146 (10) |
| Distance (m) | PTSD+Bar vs Control | ANOVA | <0.001 | t-test | 0.047 | ***** | 17.909 ± 3.471 (21) | 15.569 ± 1.009 (10) |
| Speed (m/s) | Ctrl+IHT vs Control | ANOVA | 0.003 | t-test | <0.001 | ******* | 0.028 ± 0.007 (20) | 0.017 ± 0.006 (10) |
| Speed (m/s) | PTSD+IHT vs Ctrl+IHT | ANOVA | 0.003 | t-test | 0.002 | ****** | 0.017 ± 0.006 (10) | 0.034 ± 0.012 (10) |
| Freezing episodes (n) | PTSD+CoCl₂ vs Control | ANOVA | <0.001 | t-test | 0.007 | ****** | 11.524 ± 7.236 (21) | 20.000 ± 8.955 (11) |
| Freezing episodes (n) | Ctrl+IHT vs Control | ANOVA | <0.001 | t-test | <0.001 | ******* | 11.524 ± 7.236 (21) | 28.182 ± 9.516 (11) |
| Freezing episodes (n) | PTSD+IHT vs Ctrl+IHT | ANOVA | <0.001 | t-test | <0.001 | ******* | 28.182 ± 9.516 (11) | 11.700 ± 4.084 (10) |
| Freezing episodes (n) | PTSD+Bar vs Control | ANOVA | <0.001 | t-test | 0.020 | ***** | 11.524 ± 7.236 (21) | 17.250 ± 4.634 (12) |
| Freezing episodes (n) | Ctrl+Bar vs Control | ANOVA | <0.001 | t-test | 0.029 | ***** | 11.524 ± 7.236 (21) | 17.091 ± 4.847 (11) |
| Freezing time (s) | PTSD+CoCl₂ vs Control | Kruskal | <0.001 | MWU | 0.028 | ***** | 41.986 ± 34.089 (21) | 69.073 ± 33.698 (11) |
| Freezing time (s) | Ctrl+IHT vs Control | Kruskal | <0.001 | MWU | <0.001 | ******* | 41.986 ± 34.089 (21) | 119.900 ± 46.345 (12) |
| Freezing time (s) | PTSD+IHT vs Ctrl+IHT | Kruskal | <0.001 | MWU | <0.001 | ******* | 119.900 ± 46.345 (12) | 30.250 ± 8.636 (8) |
| Freezing time (s) | PTSD+Bar vs Control | Kruskal | <0.001 | MWU | 0.035 | ***** | 41.986 ± 34.089 (21) | 60.342 ± 16.658 (12) |
| Freezing time (s) | Ctrl+Bar vs Control | Kruskal | <0.001 | MWU | 0.037 | ***** | 41.986 ± 34.089 (21) | 60.673 ± 21.863 (11) |
| Center entries (n) | PTSD+CoCl₂ vs Control | ANOVA | 0.006 | t-test | 0.032 | ***** | 13.545 ± 7.645 (22) | 7.900 ± 2.726 (10) |
| Center entries (n) | Ctrl+IHT vs Control | ANOVA | 0.006 | t-test | 0.002 | ****** | 13.545 ± 7.645 (22) | 5.545 ± 3.236 (11) |
| Corner entries (n) | Ctrl+CoCl₂ vs Control | ANOVA | 0.010 | t-test | 0.041 | ***** | 37.000 ± 20.281 (22) | 51.000 ± 13.698 (12) |
| Corner entries (n) | PTSD+IHT vs Control | ANOVA | 0.010 | t-test | 0.024 | ***** | 37.000 ± 20.281 (22) | 55.600 ± 20.945 (10) |
| Corner entries (n) | PTSD+IHT vs Ctrl+IHT | ANOVA | 0.010 | t-test | 0.017 | ***** | 37.455 ± 8.982 (11) | 55.600 ± 20.945 (10) |
| Corner entries (n) | PTSD+Bar vs Control | ANOVA | 0.010 | t-test | 0.020 | ***** | 37.000 ± 20.281 (22) | 52.083 ± 8.328 (12) |
| Corner time (s) | PTSD+CoCl₂ vs Control | Kruskal | <0.001 | MWU | 0.011 | ***** | 211.323 ± 168.908 (22) | 397.560 ± 54.182 (10) |
| Corner time (s) | Ctrl+CoCl₂ vs Control | Kruskal | <0.001 | MWU | 0.018 | ***** | 211.323 ± 168.908 (22) | 373.067 ± 71.192 (12) |
| Corner time (s) | PTSD+IHT vs Control | Kruskal | <0.001 | MWU | 0.024 | ***** | 211.323 ± 168.908 (22) | 388.562 ± 27.847 (8) |
| Corner time (s) | Ctrl+IHT vs Control | Kruskal | <0.001 | MWU | <0.001 | ******* | 211.323 ± 168.908 (22) | 446.408 ± 50.844 (12) |
| Corner time (s) | PTSD+IHT vs Ctrl+IHT | Kruskal | <0.001 | MWU | 0.020 | ***** | 446.408 ± 50.844 (12) | 388.562 ± 27.847 (8) |
| Corner time (s) | PTSD+Bar vs Control | Kruskal | <0.001 | MWU | 0.001 | ******* | 211.323 ± 168.908 (22) | 422.618 ± 48.446 (11) |
| Corner time (s) | Ctrl+Bar vs Control | Kruskal | <0.001 | MWU | 0.002 | ****** | 211.323 ± 168.908 (22) | 410.200 ± 66.748 (12) |
| Side time (s) | PTSD+IHT vs Ctrl+IHT | ANOVA | 0.104 | t-test | 0.008 | ****** | 138.600 ± 44.322 (12) | 193.737 ± 32.695 (8) |

**Supplementary Table S3. Dark-Light Box (DLB) — all significant pairwise group comparisons.**

*Same statistical workflow as Table S1.*

| **Parameter** | **Comparison** | **Overall test** | **p (overall)** | **Post-hoc** | **p (post-hoc)** | **Sig** | **Group 1: M±SD (n)** | **Group 2: M±SD (n)** |
| --- | --- | --- | --- | --- | --- | --- | --- | --- |
| Light zone entries (n) | PTSD+CoCl₂ vs Control | Kruskal | 0.001 | MWU | 0.018 | ***** | 2.333 ± 1.523 (24) | 1.222 ± 0.441 (9) |
| Light zone entries (n) | PTSD+CoCl₂ vs Ctrl+CoCl₂ | Kruskal | 0.001 | MWU | 0.012 | ***** | 3.083 ± 1.975 (12) | 1.222 ± 0.441 (9) |
| Light zone entries (n) | PTSD+IHT vs Control | Kruskal | 0.001 | MWU | 0.001 | ****** | 2.333 ± 1.523 (24) | 4.900 ± 2.283 (10) |
| Time in light (s) | PTSD+IHT vs Control | Kruskal | <0.001 | MWU | 0.031 | ***** | 34.500 ± 22.959 (22) | 59.100 ± 30.120 (10) |
| Time in light (s) | PTSD+IHT vs Ctrl+IHT | Kruskal | <0.001 | MWU | 0.009 | ****** | 25.000 ± 15.937 (11) | 59.100 ± 30.120 (10) |
| Time in light (s) | PTSD+Bar vs Control | Kruskal | <0.001 | MWU | <0.001 | ******* | 34.500 ± 22.959 (22) | 247.167 ± 32.431 (6) |
| Time in light (s) | Ctrl+Bar vs Control | Kruskal | <0.001 | MWU | <0.001 | ******* | 34.500 ± 22.959 (22) | 235.333 ± 38.769 (9) |
| Time in dark (s) | PTSD+Bar vs Control | Kruskal | <0.001 | MWU | <0.001 | ******* | 403.625 ± 173.220 (24) | 52.833 ± 32.431 (6) |
| Time in dark (s) | Ctrl+Bar vs Control | Kruskal | <0.001 | MWU | <0.001 | ******* | 403.625 ± 173.220 (24) | 59.500 ± 40.037 (10) |
| Curiosity (n) | PTSD+CoCl₂ vs Control | Kruskal | <0.001 | MWU | 0.029 | ***** | 2.875 ± 1.702 (24) | 4.455 ± 1.809 (11) |
| Curiosity (n) | Ctrl+CoCl₂ vs Control | Kruskal | <0.001 | MWU | <0.001 | ******* | 2.875 ± 1.702 (24) | 6.000 ± 1.809 (12) |
| Curiosity (n) | PTSD+IHT vs Control | Kruskal | <0.001 | MWU | <0.001 | ******* | 2.875 ± 1.702 (24) | 6.900 ± 1.969 (10) |
| Curiosity (n) | Ctrl+IHT vs Control | Kruskal | <0.001 | MWU | 0.005 | ****** | 2.875 ± 1.702 (24) | 5.300 ± 2.263 (10) |

**Supplementary Table S4. qPCR results — medial prefrontal cortex (mPFC).**

*All 25 a priori pairwise comparisons per gene. Values: Mean ± SEM (n). Significance after Benjamini-Hochberg FDR correction independently per gene. *p<0.05, **p<0.01, ***p<0.001, ****p<0.0001, ns — not significant.*

| **Comparison** | **HIF-1α** | **HIF-2α** | **HIF-3α** | **PACAP** | **PAI-1** |
| --- | --- | --- | --- | --- | --- |
| PTSD vs Control | 1.609±0.387 (8) ns | 1.139±0.407 (8) ns | 7.227±4.311 (8) ns | 1.781±1.192 (8) ns | 1.838±0.789 (8) ns |
| Control+CoCl₂ vs Control | 0.129±0.045 (9) ** | 0.093±0.039 (8) *** | 0.134±0.047 (9) ns | 0.017±0.002 (8) ** | 0.010±0.003 (9) *** |
| Control+IHT vs Control | 0.084±0.021 (8) ** | 0.014±0.001 (9) *** | 0.052±0.007 (9) * | 0.035±0.009 (9) * | 0.008±0.003 (8) *** |
| Control+Bar vs Control | 0.274±0.128 (8) * | 0.037±0.009 (7) *** | 0.371±0.297 (8) ns | 0.501±0.363 (8) ns | 0.005±0.002 (7) *** |
| PTSD+CoCl₂ vs PTSD | 0.272±0.186 (9) ** | 0.029±0.007 (9) *** | 0.065±0.008 (8) *** | 0.146±0.109 (9) * | 0.004±0.001 (8) *** |
| PTSD+IHT vs PTSD | 0.178±0.143 (8) ** | 0.013±0.001 (8) *** | 0.011±0.002 (8) *** | 0.021±0.005 (8) ** | 0.002±0.001 (8) *** |
| PTSD+Bar vs PTSD | 0.044±0.008 (8) ** | 0.014±0.003 (9) *** | 0.009±0.001 (9) *** | 0.011±0.002 (8) ** | 0.003±0.001 (9) *** |
| PTSD+CoCl₂ vs Control | 0.272±0.186 (9) ** | 0.029±0.007 (9) *** | 0.065±0.008 (8) * | 0.146±0.109 (9) * | 0.004±0.001 (8) *** |
| PTSD+CoCl₂ vs Control+CoCl₂ | 0.272±0.186 (9) ns | 0.029±0.007 (9) ns | 0.065±0.008 (8) ns | 0.146±0.109 (9) ns | 0.004±0.001 (8) ns |
| PTSD+IHT vs Control | 0.178±0.143 (8) ** | 0.013±0.001 (8) *** | 0.011±0.002 (8) * | 0.021±0.005 (8) ** | 0.002±0.001 (8) *** |
| PTSD+IHT vs Control+IHT | 0.178±0.143 (8) ns | 0.013±0.001 (8) ns | 0.011±0.002 (8) *** | 0.021±0.005 (8) ns | 0.002±0.001 (8) ns |
| PTSD+Bar vs Control | 0.044±0.008 (8) ** | 0.014±0.003 (9) *** | 0.009±0.001 (9) * | 0.011±0.002 (8) ** | 0.003±0.001 (9) *** |
| PTSD+Bar vs Control+Bar | 0.044±0.008 (8) ** | 0.014±0.003 (9) ** | 0.009±0.001 (9) ** | 0.011±0.002 (8) * | 0.003±0.001 (9) ns |
| Control+IHT vs Control+CoCl₂ | 0.084±0.021 (8) ns | 0.014±0.001 (9) * | 0.052±0.007 (9) ns | 0.035±0.009 (9) ns | 0.008±0.003 (8) ns |
| Control+Bar vs Control+CoCl₂ | 0.274±0.128 (8) ns | 0.037±0.009 (7) ns | 0.371±0.297 (8) ns | 0.501±0.363 (8) ns | 0.005±0.002 (7) ns |
| Control+Bar vs Control+IHT | 0.274±0.128 (8) ns | 0.037±0.009 (7) ** | 0.371±0.297 (8) ns | 0.501±0.363 (8) ns | 0.005±0.002 (7) ns |
| PTSD+IHT vs PTSD+CoCl₂ | 0.178±0.143 (8) ns | 0.013±0.001 (8) * | 0.011±0.002 (8) *** | 0.021±0.005 (8) ns | 0.002±0.001 (8) ns |
| PTSD+Bar vs PTSD+CoCl₂ | 0.044±0.008 (8) ns | 0.014±0.003 (9) * | 0.009±0.001 (9) *** | 0.011±0.002 (8) ns | 0.003±0.001 (9) ns |
| PTSD+Bar vs PTSD+IHT | 0.044±0.008 (8) ns | 0.014±0.003 (9) ns | 0.009±0.001 (9) ns | 0.011±0.002 (8) ns | 0.003±0.001 (9) ns |
| PTSD+IHT vs Control+CoCl₂ | 0.178±0.143 (8) ns | 0.013±0.001 (8) * | 0.011±0.002 (8) *** | 0.021±0.005 (8) ns | 0.002±0.001 (8) ** |
| PTSD+Bar vs Control+CoCl₂ | 0.044±0.008 (8) ns | 0.014±0.003 (9) * | 0.009±0.001 (9) ** | 0.011±0.002 (8) ns | 0.003±0.001 (9) * |
| Control+IHT vs PTSD+CoCl₂ | 0.084±0.021 (8) ns | 0.014±0.001 (9) ** | 0.052±0.007 (9) ns | 0.035±0.009 (9) ns | 0.008±0.003 (8) ns |
| Control+Bar vs PTSD+CoCl₂ | 0.274±0.128 (8) ns | 0.037±0.009 (7) ns | 0.371±0.297 (8) ns | 0.501±0.363 (8) ns | 0.005±0.002 (7) ns |
| PTSD+Bar vs Control+IHT | 0.044±0.008 (8) ns | 0.014±0.003 (9) ns | 0.009±0.001 (9) ** | 0.011±0.002 (8) ns | 0.003±0.001 (9) ns |
| Control+Bar vs PTSD+IHT | 0.274±0.128 (8) * | 0.037±0.009 (7) *** | 0.371±0.297 (8) ** | 0.501±0.363 (8) ns | 0.005±0.002 (7) ns |

**Supplementary Table S5. qPCR results — hippocampus.**

*All 25 a priori pairwise comparisons per gene. Values: Mean ± SEM (n). Significance after Benjamini-Hochberg FDR correction independently per gene. *p<0.05, **p<0.01, ***p<0.001, ****p<0.0001, ns — not significant.*

| **Comparison** | **HIF-1α** | **HIF-2α** | **HIF-3α** | **PACAP** | **PAI-1** |
| --- | --- | --- | --- | --- | --- |
| PTSD vs Control | 2.783±0.655 (9) * | 2.048±0.444 (9) ns | 32.489±15.796 (8) ns | 3.636±1.723 (9) ns | 2.412±0.764 (8) ns |
| Control+CoCl₂ vs Control | 0.086±0.016 (9) ** | 0.036±0.008 (9) ** | 0.270±0.018 (8) ns | 0.025±0.006 (9) ** | 0.003±0.001 (8) *** |
| Control+IHT vs Control | 0.084±0.025 (9) ** | 0.025±0.002 (9) ** | 0.193±0.024 (9) ns | 0.035±0.007 (9) ** | 0.003±0.001 (9) *** |
| Control+Bar vs Control | 0.105±0.018 (8) *** | 0.047±0.007 (8) *** | 0.222±0.094 (9) ns | 0.079±0.028 (9) ** | 0.013±0.006 (9) *** |
| PTSD+CoCl₂ vs PTSD | 0.121±0.044 (9) ** | 0.029±0.003 (9) ** | 0.207±0.024 (9) *** | 0.036±0.013 (9) ** | 0.009±0.004 (9) *** |
| PTSD+IHT vs PTSD | 0.050±0.011 (8) *** | 0.022±0.001 (8) *** | 0.046±0.014 (9) *** | 0.022±0.005 (8) ** | 0.005±0.002 (9) *** |
| PTSD+Bar vs PTSD | 0.031±0.010 (9) ** | 0.015±0.004 (9) ** | 0.040±0.013 (9) *** | 0.018±0.007 (9) ** | 0.004±0.001 (9) *** |
| PTSD+CoCl₂ vs Control | 0.121±0.044 (9) ** | 0.029±0.003 (9) ** | 0.207±0.024 (9) ns | 0.036±0.013 (9) ** | 0.009±0.004 (9) *** |
| PTSD+CoCl₂ vs Control+CoCl₂ | 0.121±0.044 (9) ns | 0.029±0.003 (9) ns | 0.207±0.024 (9) ns | 0.036±0.013 (9) ns | 0.009±0.004 (9) ns |
| PTSD+IHT vs Control | 0.050±0.011 (8) *** | 0.022±0.001 (8) *** | 0.046±0.014 (9) ** | 0.022±0.005 (8) ** | 0.005±0.002 (9) *** |
| PTSD+IHT vs Control+IHT | 0.050±0.011 (8) ns | 0.022±0.001 (8) ns | 0.046±0.014 (9) ** | 0.022±0.005 (8) ns | 0.005±0.002 (9) ns |
| PTSD+Bar vs Control | 0.031±0.010 (9) ** | 0.015±0.004 (9) ** | 0.040±0.013 (9) ** | 0.018±0.007 (9) ** | 0.004±0.001 (9) *** |
| PTSD+Bar vs Control+Bar | 0.031±0.010 (9) ** | 0.015±0.004 (9) ** | 0.040±0.013 (9) * | 0.018±0.007 (9) * | 0.004±0.001 (9) ns |
| Control+IHT vs Control+CoCl₂ | 0.084±0.025 (9) ns | 0.025±0.002 (9) ns | 0.193±0.024 (9) ns | 0.035±0.007 (9) ns | 0.003±0.001 (9) ns |
| Control+Bar vs Control+CoCl₂ | 0.105±0.018 (8) ns | 0.047±0.007 (8) ns | 0.222±0.094 (9) ns | 0.079±0.028 (9) * | 0.013±0.006 (9) ns |
| Control+Bar vs Control+IHT | 0.105±0.018 (8) ns | 0.047±0.007 (8) * | 0.222±0.094 (9) ns | 0.079±0.028 (9) ns | 0.013±0.006 (9) * |
| PTSD+IHT vs PTSD+CoCl₂ | 0.050±0.011 (8) ns | 0.022±0.001 (8) * | 0.046±0.014 (9) ** | 0.022±0.005 (8) ns | 0.005±0.002 (9) ns |
| PTSD+Bar vs PTSD+CoCl₂ | 0.031±0.010 (9) * | 0.015±0.004 (9) ns | 0.040±0.013 (9) ** | 0.018±0.007 (9) ns | 0.004±0.001 (9) ns |
| PTSD+Bar vs PTSD+IHT | 0.031±0.010 (9) ns | 0.015±0.004 (9) ns | 0.040±0.013 (9) ns | 0.018±0.007 (9) ns | 0.004±0.001 (9) ns |
| PTSD+IHT vs Control+CoCl₂ | 0.050±0.011 (8) ns | 0.022±0.001 (8) ns | 0.046±0.014 (9) *** | 0.022±0.005 (8) ns | 0.005±0.002 (9) ns |
| PTSD+Bar vs Control+CoCl₂ | 0.031±0.010 (9) * | 0.015±0.004 (9) ns | 0.040±0.013 (9) *** | 0.018±0.007 (9) ns | 0.004±0.001 (9) ns |
| Control+IHT vs PTSD+CoCl₂ | 0.084±0.025 (9) ns | 0.025±0.002 (9) ns | 0.193±0.024 (9) ns | 0.035±0.007 (9) ns | 0.003±0.001 (9) ns |
| Control+Bar vs PTSD+CoCl₂ | 0.105±0.018 (8) ns | 0.047±0.007 (8) ns | 0.222±0.094 (9) ns | 0.079±0.028 (9) ns | 0.013±0.006 (9) ns |
| PTSD+Bar vs Control+IHT | 0.031±0.010 (9) ns | 0.015±0.004 (9) ns | 0.040±0.013 (9) ** | 0.018±0.007 (9) ns | 0.004±0.001 (9) ns |
| Control+Bar vs PTSD+IHT | 0.105±0.018 (8) * | 0.047±0.007 (8) ** | 0.222±0.094 (9) ns | 0.079±0.028 (9) * | 0.013±0.006 (9) ns |

**Supplementary Table S6. PAI-1 raw Ct values across all groups, with corresponding no-template control (NTC) Ct values.**

*Individual sample PAI-1 Ct values are reported per group with mean ± SEM. NTC values represent no-template control reactions monitored on the qPCR plates. The clean NTC measurement at Ct ~38 provides >7-cycle separation from the lowest sample Ct (~27), confirming that all sample signals are clearly separated from background. The second NTC value (Ct ~28) reflects late, non-specific amplification monitored as part of plate quality control and is not a contamination of the target signal.*

| **Region** | **Group** | **n** | **Mean Ct ± SEM** | **Range (min–max)** | **Individual Ct values** |
| --- | --- | --- | --- | --- | --- |
| **mPFC** | Control | 7 | 30.56 ± 0.32 | 29.17 – 31.45 | 31.18, 30.57, 31.45, 29.42, 29.17, 31.06, 31.07 |
|  | PTSD | 8 | 30.30 ± 0.51 | 27.53 – 32.76 | 31.10, 31.12, 30.26, 32.76, 30.59, 29.22, 29.78, 27.53 |
|  | Control+CoCl₂ | 9 | 29.51 ± 0.28 | 28.28 – 30.81 | 30.29, 28.95, 30.10, 28.60, 29.99, 28.28, 28.66, 30.81, 29.92 |
|  | PTSD+CoCl₂ | 8 | 30.76 ± 0.26 | 29.14 – 31.68 | 30.65, 29.14, 31.00, 31.68, 31.57, 30.47, 30.60, 30.99 |
|  | Control+IHT | 9 | 29.78 ± 0.36 | 28.05 – 31.12 | 28.05, 29.66, 30.05, 28.34, 30.84, 31.12, 30.58, 28.81, 30.59 |
|  | PTSD+IHT | 9 | 29.61 ± 0.40 | 27.19 – 31.04 | 30.40, 29.44, 29.66, 31.04, 27.19, 29.65, 28.02, 30.80, 30.31 |
|  | Control+Bar | 9 | 29.00 ± 0.38 | 27.86 – 31.96 | 31.96, 28.23, 29.59, 28.83, 28.74, 27.86, 28.79, 28.38, 28.65 |
|  | PTSD+Bar | 9 | 29.62 ± 0.19 | 28.51 – 30.20 | 29.95, 29.11, 30.06, 28.51, 30.05, 30.14, 30.20, 29.55, 29.04 |
| **Hippocampus** | Control | 6 | 29.85 ± 0.48 | 27.97 – 31.55 | 30.43, 30.05, 28.75, 30.35, 31.55, 27.97 |
|  | PTSD | 6 | 29.56 ± 0.78 | 26.77 – 31.77 | 31.54, 31.77, 27.56, 30.66, 29.04, 26.77 |
|  | Control+CoCl₂ | 8 | 30.98 ± 0.20 | 29.96 – 31.67 | 31.22, 31.01, 30.95, 29.96, 31.28, 31.67, 31.47, 30.24 |
|  | PTSD+CoCl₂ | 8 | 30.72 ± 0.60 | 28.04 – 33.49 | 28.04, 33.49, 29.86, 31.14, 31.51, 31.07, 32.04, 28.61 |
|  | Control+IHT | 8 | 30.94 ± 0.30 | 28.92 – 31.76 | 31.35, 31.29, 31.55, 30.46, 31.28, 31.76, 28.92, 30.94 |
|  | PTSD+IHT | 7 | 29.72 ± 0.32 | 28.04 – 30.78 | 30.78, 29.38, 29.97, 28.04, 29.30, 30.28, 30.32 |
|  | Control+Bar | 7 | 29.37 ± 0.35 | 27.93 – 30.48 | 29.48, 30.33, 27.93, 30.48, 29.21, 28.17, 30.00 |
|  | PTSD+Bar | 8 | 29.77 ± 0.35 | 28.02 – 31.78 | 29.94, 31.78, 29.72, 30.13, 29.98, 28.02, 29.61, 28.97 |
| **NTC** | no-template (plate QC) | 1 | 38.06 | — | *clean — >7-cycle separation from lowest sample Ct* |
| **NTC** | no-template (plate QC) | 1 | 28.36 | — | *late non-specific signal monitored as plate QC* |

**Supplementary Table S7. ΔCt outliers removed by Grubbs’ test (α=0.05, iterative).**

*Delta-Ct outliers detected per group×tissue×gene subset prior to ΔΔCt/[C]rel calculation. Removal performed iteratively (largest deviation removed first, test repeated) until no further outlier met the Grubbs’ criterion.*

| **Tissue** | **Gene** | **Group + treatment** | **Removed ΔCt value** | **n before removal** |
| --- | --- | --- | --- | --- |
| Hippocampus | HIF-1α | Control+Bar | 5.1506 | 9 |
| Hippocampus | HIF-2α | Control+Bar | 2.0915 | 9 |
| mPFC | HIF-1α | Control+Bar | -8.4013 | 9 |
| mPFC | HIF-2α | Control+Bar | -11.0471 | 9 |
| mPFC | HIF-2α | Control+Bar | 1.6803 | 9 |
| mPFC | HIF-3α | Control+Bar | -7.9779 | 9 |
| mPFC | PACAP | Control+Bar | -8.7660 | 9 |
| mPFC | PAI-1 | Control+Bar | -4.0186 | 9 |
| mPFC | PAI-1 | Control+Bar | 5.8994 | 9 |
| Hippocampus | HIF-3α | Control+CoCl₂ | 10.0794 | 9 |
| Hippocampus | PAI-1 | Control+CoCl₂ | 5.0967 | 9 |
| mPFC | PACAP | Control+CoCl₂ | 7.1392 | 9 |
| mPFC | HIF-1α | Control+IHT | 16.2392 | 9 |
| Hippocampus | PAI-1 | Control | -2.0798 | 9 |
| mPFC | HIF-1α | Control | -3.7042 | 9 |
| mPFC | HIF-2α | Control | -7.4952 | 9 |
| mPFC | PAI-1 | Control | -4.0266 | 9 |
| mPFC | HIF-3α | PTSD+CoCl₂ | 6.6980 | 9 |
| mPFC | PAI-1 | PTSD+CoCl₂ | 5.8394 | 9 |
| Hippocampus | HIF-1α | PTSD+IHT | 13.6044 | 9 |
| Hippocampus | HIF-2α | PTSD+IHT | 5.2435 | 9 |
| Hippocampus | PACAP | PTSD+IHT | 15.9987 | 9 |
| mPFC | HIF-2α | PTSD+IHT | 5.4910 | 9 |
| mPFC | HIF-3α | PTSD+IHT | 5.1668 | 9 |
| mPFC | PACAP | PTSD+IHT | 3.6779 | 9 |
| mPFC | PAI-1 | PTSD+IHT | -1.9818 | 9 |
| Hippocampus | PAI-1 | PTSD | -4.9122 | 9 |

**Supplementary Table S8. Spearman correlations — gene expression vs behavioural parameters (all comparisons).**

*Group-mean [C]rel (n=7 groups per tissue/gene) correlated with group-mean behavioural parameters. Spearman rank correlation; * p<0.05, ** p<0.01, *** p<0.001, ns — not significant.*

| **Tissue** | **Gene** | **Behavioural parameter** | **n** | **r** | **p** | **Sig** |
| --- | --- | --- | --- | --- | --- | --- |
| Hippocampus | HIF-1α | Center time OF (s) | 7 | 0.7143 | 0.0713 | ns |
| Hippocampus | HIF-1α | Corner time OF (s) | 7 | -0.5357 | 0.2152 | ns |
| Hippocampus | HIF-1α | Freezing episodes EPM (n) | 7 | -0.0357 | 0.9394 | ns |
| Hippocampus | HIF-1α | Freezing time OF (s) | 7 | 0.0714 | 0.8790 | ns |
| Hippocampus | HIF-1α | Light zone entries DLB (n) | 7 | -0.9286 | 0.0025 | ** |
| Hippocampus | HIF-1α | Open arm time EPM (s) | 7 | -0.2500 | 0.5887 | ns |
| Hippocampus | HIF-1α | Open head dips EPM (n) | 7 | 0.1071 | 0.8192 | ns |
| Hippocampus | HIF-1α | Time in light DLB (s) | 7 | -0.5714 | 0.1802 | ns |
| Hippocampus | HIF-2α | Center time OF (s) | 7 | 0.8214 | 0.0234 | * |
| Hippocampus | HIF-2α | Corner time OF (s) | 7 | -0.5714 | 0.1802 | ns |
| Hippocampus | HIF-2α | Freezing episodes EPM (n) | 7 | -0.2500 | 0.5887 | ns |
| Hippocampus | HIF-2α | Freezing time OF (s) | 7 | -0.0714 | 0.8790 | ns |
| Hippocampus | HIF-2α | Light zone entries DLB (n) | 7 | -0.7500 | 0.0522 | ns |
| Hippocampus | HIF-2α | Open arm time EPM (s) | 7 | -0.0714 | 0.8790 | ns |
| Hippocampus | HIF-2α | Open head dips EPM (n) | 7 | 0.1071 | 0.8192 | ns |
| Hippocampus | HIF-2α | Time in light DLB (s) | 7 | -0.2857 | 0.5345 | ns |
| Hippocampus | HIF-3α | Center time OF (s) | 7 | 0.8571 | 0.0137 | * |
| Hippocampus | HIF-3α | Corner time OF (s) | 7 | -0.6786 | 0.0938 | ns |
| Hippocampus | HIF-3α | Freezing episodes EPM (n) | 7 | -0.1786 | 0.7017 | ns |
| Hippocampus | HIF-3α | Freezing time OF (s) | 7 | -0.1429 | 0.7599 | ns |
| Hippocampus | HIF-3α | Light zone entries DLB (n) | 7 | -0.7143 | 0.0713 | ns |
| Hippocampus | HIF-3α | Open arm time EPM (s) | 7 | -0.1071 | 0.8192 | ns |
| Hippocampus | HIF-3α | Open head dips EPM (n) | 7 | 0.0357 | 0.9394 | ns |
| Hippocampus | HIF-3α | Time in light DLB (s) | 7 | -0.3571 | 0.4316 | ns |
| Hippocampus | PACAP | Center time OF (s) | 7 | 0.5714 | 0.1802 | ns |
| Hippocampus | PACAP | Corner time OF (s) | 7 | -0.3214 | 0.4821 | ns |
| Hippocampus | PACAP | Freezing episodes EPM (n) | 7 | -0.2143 | 0.6445 | ns |
| Hippocampus | PACAP | Freezing time OF (s) | 7 | 0.1786 | 0.7017 | ns |
| Hippocampus | PACAP | Light zone entries DLB (n) | 7 | -0.8214 | 0.0234 | * |
| Hippocampus | PACAP | Open arm time EPM (s) | 7 | -0.1786 | 0.7017 | ns |
| Hippocampus | PACAP | Open head dips EPM (n) | 7 | 0.1071 | 0.8192 | ns |
| Hippocampus | PACAP | Time in light DLB (s) | 7 | -0.4643 | 0.2939 | ns |
| Hippocampus | PAI-1 | Center time OF (s) | 7 | 0.6071 | 0.1482 | ns |
| Hippocampus | PAI-1 | Corner time OF (s) | 7 | -0.5000 | 0.2532 | ns |
| Hippocampus | PAI-1 | Freezing episodes EPM (n) | 7 | -0.3214 | 0.4821 | ns |
| Hippocampus | PAI-1 | Freezing time OF (s) | 7 | -0.3214 | 0.4821 | ns |
| Hippocampus | PAI-1 | Light zone entries DLB (n) | 7 | -0.5714 | 0.1802 | ns |
| Hippocampus | PAI-1 | Open arm time EPM (s) | 7 | 0.3571 | 0.4316 | ns |
| Hippocampus | PAI-1 | Open head dips EPM (n) | 7 | 0.7143 | 0.0713 | ns |
| Hippocampus | PAI-1 | Time in light DLB (s) | 7 | 0.0357 | 0.9394 | ns |
| mPFC | HIF-1α | Center time OF (s) | 7 | 0.6429 | 0.1194 | ns |
| mPFC | HIF-1α | Corner time OF (s) | 7 | -0.6071 | 0.1482 | ns |
| mPFC | HIF-1α | Freezing episodes EPM (n) | 7 | -0.4286 | 0.3374 | ns |
| mPFC | HIF-1α | Freezing time OF (s) | 7 | -0.2500 | 0.5887 | ns |
| mPFC | HIF-1α | Light zone entries DLB (n) | 7 | -0.6786 | 0.0938 | ns |
| mPFC | HIF-1α | Open arm time EPM (s) | 7 | 0.1786 | 0.7017 | ns |
| mPFC | HIF-1α | Open head dips EPM (n) | 7 | 0.4643 | 0.2939 | ns |
| mPFC | HIF-1α | Time in light DLB (s) | 7 | -0.2500 | 0.5887 | ns |
| mPFC | HIF-2α | Center time OF (s) | 7 | 0.8929 | 0.0068 | ** |
| mPFC | HIF-2α | Corner time OF (s) | 7 | -0.5714 | 0.1802 | ns |
| mPFC | HIF-2α | Freezing episodes EPM (n) | 7 | 0.0000 | 1.0000 | ns |
| mPFC | HIF-2α | Freezing time OF (s) | 7 | -0.0357 | 0.9394 | ns |
| mPFC | HIF-2α | Light zone entries DLB (n) | 7 | -0.7500 | 0.0522 | ns |
| mPFC | HIF-2α | Open arm time EPM (s) | 7 | -0.1786 | 0.7017 | ns |
| mPFC | HIF-2α | Open head dips EPM (n) | 7 | 0.0000 | 1.0000 | ns |
| mPFC | HIF-2α | Time in light DLB (s) | 7 | -0.2857 | 0.5345 | ns |
| mPFC | HIF-3α | Center time OF (s) | 7 | 0.8214 | 0.0234 | * |
| mPFC | HIF-3α | Corner time OF (s) | 7 | -0.5714 | 0.1802 | ns |
| mPFC | HIF-3α | Freezing episodes EPM (n) | 7 | -0.2500 | 0.5887 | ns |
| mPFC | HIF-3α | Freezing time OF (s) | 7 | -0.0714 | 0.8790 | ns |
| mPFC | HIF-3α | Light zone entries DLB (n) | 7 | -0.7500 | 0.0522 | ns |
| mPFC | HIF-3α | Open arm time EPM (s) | 7 | -0.0714 | 0.8790 | ns |
| mPFC | HIF-3α | Open head dips EPM (n) | 7 | 0.1071 | 0.8192 | ns |
| mPFC | HIF-3α | Time in light DLB (s) | 7 | -0.2857 | 0.5345 | ns |
| mPFC | PACAP | Center time OF (s) | 7 | 0.4286 | 0.3374 | ns |
| mPFC | PACAP | Corner time OF (s) | 7 | -0.2857 | 0.5345 | ns |
| mPFC | PACAP | Freezing episodes EPM (n) | 7 | -0.3571 | 0.4316 | ns |
| mPFC | PACAP | Freezing time OF (s) | 7 | 0.1071 | 0.8192 | ns |
| mPFC | PACAP | Light zone entries DLB (n) | 7 | -0.7143 | 0.0713 | ns |
| mPFC | PACAP | Open arm time EPM (s) | 7 | -0.0357 | 0.9394 | ns |
| mPFC | PACAP | Open head dips EPM (n) | 7 | 0.2500 | 0.5887 | ns |
| mPFC | PACAP | Time in light DLB (s) | 7 | -0.4286 | 0.3374 | ns |
| mPFC | PAI-1 | Center time OF (s) | 7 | 0.6429 | 0.1194 | ns |
| mPFC | PAI-1 | Corner time OF (s) | 7 | -0.3929 | 0.3833 | ns |
| mPFC | PAI-1 | Freezing episodes EPM (n) | 7 | -0.0714 | 0.8790 | ns |
| mPFC | PAI-1 | Freezing time OF (s) | 7 | 0.0714 | 0.8790 | ns |
| mPFC | PAI-1 | Light zone entries DLB (n) | 7 | -0.5357 | 0.2152 | ns |
| mPFC | PAI-1 | Open arm time EPM (s) | 7 | -0.2143 | 0.6445 | ns |
| mPFC | PAI-1 | Open head dips EPM (n) | 7 | -0.1786 | 0.7017 | ns |
| mPFC | PAI-1 | Time in light DLB (s) | 7 | -0.3929 | 0.3833 | ns |
